# A Novel Molecular Method for Simultaneous Identification of *Vibrio parahaemolyticus* 57 K-Serogroups Using Probe Melting Curve Analysis

**DOI:** 10.3389/fcimb.2021.594808

**Published:** 2021-02-26

**Authors:** Linying Lu, Minxu Li, Yinghui Li, Min Jiang, Yixiang Jiang, Xiaolu Shi, Le Zuo, Lei Wang, Shengzhe Bian, Yaqun Qiu, Rui Cai, Yiqun Liao, Qingge Li, Liqiang Li, Qinghua Hu

**Affiliations:** ^1^ School of Public Health, University of South China, Hengyang, China; ^2^ Shenzhen Center for Disease Control and Prevention, Shenzhen, China; ^3^ School of Public Health (Shenzhen), Sun Yat-sen University, Guangzhou, China; ^4^ Shenzhen Key Laboratory of Unknown Pathogen Identification, BGI-Shenzhen, Shenzhen, China; ^5^ School of Life Sciences, Xiamen University, Xiamen, China

**Keywords:** *Vibrio parahaemolyticus*, molecular identification, K-serogroups, vibriosis surveillance, multiplex ligation reaction based on probe melting curve analysis

## Abstract

The serotyping of *Vibrio parahaemolyticus*, which is crucial to the surveillance and detection of outbreaks of vibriosis infection, has been widely used in many countries. In this study, we developed a molecular assay, named multiplex ligation reaction based on probe melting curve analysis (MLMA), for simultaneous identification of *V. parahaemolyticus* 57 K-serogroups. Based on the previous genomes of 418 strains including 39 K-serogroups and the 18 K-serogroups sequences from public databases, we obtained 57 K-serogroups specific gene sequences for designing primers and probes. The developed MLMA assay for identifying the *V. parahaemolyticus* 57 K-serogroups showed high reproducibility, with the intra- and inter-assay standard deviations and coefficients of variation of no more than 1°C and 1%, respectively. The limit of detection for all gene targets ranged from 0.1 to 1.0 ng/µl. We validated the MLMA assay with a double-blind test identifying 595 *V. parahaemolyticus* isolates using conventional serotyping methods for comparison. The results showed the kappa value between the MLMA assay and the traditional serological method was 0.936 and that there was a 96.97% consistency rate with conventional serotyping methods for all detected isolates. Additionally, five rare K-serogroups were identified using the MLMA assay, as well as 18 strains that could not be identified using the traditional serotyping method. Thus, the MLMA assay provides a rapid, robust, and promising tool for the molecular serotyping of *V. parahaemolyticus* K-serogroups and has the potential application to the detection of outbreaks and surveillance of *V. parahaemolyticus* infection.

## Introduction

*Vibrio parahaemolyticus* is a Gram-negative, motile bacterium commonly found in marine and estuarine environments worldwide (Letchumanan et al., 2014; Wang et al., 2015). Investigation of *V. parahaemolyticus* infection has revealed that raw or undercooked seafood contaminated by *V. parahaemolyticus* strains is commonly responsible for acute gastroenteritis, and that in rare cases such as wound infection, ear infection or septicemia (Letchumanan et al., 2014). Between 2011 and 2016, 790 outbreaks of foodborne diseases were reported in more than 703 hospitals of China, causing 13,013 individuals to become ill. Of these cases, *V. parahaemolyticus* was the most common pathogen being responsible for foodborne disease outbreaks that caused most cases reported (42.3%). Therefore, *V. parahaemolyticus* is recognized as the leading cause of foodborne disease in China (Liu et al., 2018). Recent studies have found that pandemic *V. parahaemolyticus* strains are responsible for approximately 50%–80% of all *V. parahaemolyticus* cases in many countries, including India, Peru, Mexico and Chile (Han et al., 2019). In addition, a newly emerging shrimp disease, acute hepatopancreatic necrosis disease (AHPND), is known to be caused by strains of *V. parahaemolyticus* that contain a unique virulence plasmid. It has caused significant economic loss in global shrimp industry (Lai et al., 2015). Recent genomic analysis of 233 *V. parahaemolyticus* strains isolated from diseased shrimp, humans and environmental samples suggested that the spread of AHPND is mainly *via* horizontal transfer of the AHPND-associated plasmid, which highlighted a significant transmission route of *V. parahaemolyticus* between aquaculture and human communities (Fu et al., 2020). Thus, *V. parahaemolyticus* has become a large public health issue on a global scale, especially in coastal regions (Han et al., 2016).

*V. parahaemolyticus* is a multiserotype bacterium found in foodborne diseases or external environments that includes at least 13 O-serogroups and 71 K-serotypes (Iguchi et al., 1995; Bhuiyan et al., 2002). In 1996, the epidemiology of *V. parahaemolyticus* displayed a radical change with a new serotype, O3:K6, that appeared abruptly (Nair et al., 2007; Baker-Austin et al., 2018). The pandemic O3:K6 strains have specific genetic markers such as positivity for the thermostable direct hemolysin (*tdh*) gene, negativity for the TDH-related hemolysin (*trh*) gene and positivity for a *toxRS/new* gene (Matsumoto et al., 2000; Han et al., 2016). Since then, the serotype O3:K6 and its serovariants have spread rapidly throughout the world (Han et al., 2016). Additionally, a new emerging serotype with enhanced acid resistance, O4:KUT-recAin, has become the second most common serotype following O3:K6. In China, O4:KUT-recAin has become widespread during a short period of time (Chen et al., 2020). Accordingly, serotyping has been widely applied to provide a vital information for epidemiological investigation.

Serotyping is an essential method for identification of pathogens, foodborne disease outbreak investigations and source tracing that has been widely used to obtain an accurate biological phenotype of many pathogens. The serotyping of *V. parahaemolyticus* is generally accomplished using traditional methods based on agglutination with certain antisera. However, serological methods are limited in that they are time-consuming, expensive and labor-intensive. As understanding of antigens and their gene clusters has improved, some molecular approaches have been developed as alternatives to the traditional method. For example, polymerase chain reaction (PCR) assays are often used to identify and distinguish specific serotype. In 2012, an O-serogroup-specific PCR based assay was first used for the identification and detection of *V. parahaemolyticus* pathogens in clinical and environmental samples (Chen et al., 2012). Later, the use of the matrix-assisted laser desorption/ionization time-of-flight mass spectrometry (MALDI-TOF MS) enabled detection of a specific serotype, O4:K8, but failed to identify other serotypes (Li et al., 2018). Recently, a microsphere-based suspension array was established for the detection and identification of 55 *V. parahaemolyticus* K-serogroups based on specific genes of the capsular polysaccharide (CPS) gene cluster (Pang et al., 2019). However, these methods are limited in that they require labor-intensive post-PCR manipulations, expensive instruments, or involve the preparation of microsphere beads, which are complicated and time-consuming.

Multiplex ligation reaction based on probe melting curve analysis (MLMA) is a high-throughput, high-accuracy, low-cost method that has been successfully used for the simultaneous identification of 10 bacterial pathogens and applied to the simultaneous identification of 30 common *Salmonella* serotypes (Jiang et al., 2017; Zuo et al., 2019). In MLMA, a fluorescence signal and melting temperature (Tm) are combined and used as a virtual 2D label that enables homogenous detection of one order of magnitude more targets (Liao et al., 2013). Previously, we presented a molecular assay based on the principles of MLMA to simultaneously identify the 11 clinically most common *V. parahaemolyticus* serotypes (Li et al., 2019). In this study, to further increase the number of target K-serogroups in multiplexity, we established a three-tube system to serotype *V. parahaemolyticus* 57 K-serogroups that could be applied as an alternative for the surveillance and control of vibriosis infections.

## Materials and Methods

### Bacterial Strains

There were 3,826 strains stored that were isolated from the stool specimens of infectious diarrheal patients and food samples over 15 years (2003–2018) in Shenzhen Center for Disease Control and Prevention, 3,590 strains were K-serogroups typeable and 236 strains were K-serogroups untypeable (KUT). Among 3,826 strains, 418 strains were previously sequenced (Li et al., 2019; Bian et al., 2021). Among 418 strains, 338 strains were selected to develop the MLMA assay (**Table 1**), which included 328 strains representing 39 K-serogroups and 10 strains of untyped K-serogroups for detection of cross-reactions. In addition, 18 rare K-serogroups were incorporated into the assay using *Escherichia coli* TOP10 strains (n=18, **Table S1**) containing serogroup-specific genes cloned into a pUC57 vector (Sangon Biotech Co. Ltd., Shanghai, China). Among 3,826 strains, 595 strains which included 10% of 3,590 K-serogroups typeable strains (n=359, **Table S2**) and all 236 untypeable strains (**Table S3**) were selected to validate the MLMA assay.

**Table 1:** Reference isolates (*n* = 338) representing *V. parahaemolyticus* K-serogroups over a 12-year period (2006–2018) for the development of multiplex ligation reaction based on probe melting curve analysis (MLMA) assay.

Serotype	Number of isolates
K1	3
K3	10
K4	6
K5	1
K6	20
K8	20
K9	20
K11	6
K12	4
K13	11
K17	11
K18	3
K19	4
K20	10
K21	2
K23	2
K25	20
K28	9
K29	20
K30	3
K31	1
K32	14
K33	6
K34	10
K36	20
K37	2
K38	2
K41	9
K42	7
K44	8
K48	1
K49	1
K55	3
K56	20
K60	7
K63	4
K68	20
K69	7
K71	1
KUT	10

### Bacterial Culture, DNA Extraction, and Conventional Serotyping

All *V. parahaemolyticus* strains were revived and cultured in Vibrio chromogenic agar (Guangdong Huankai Microbial Science and Technology, Guangzhou, China) at 37°C for 12 h to isolate single colonies. Individual colonies were then selected and cultured in 3 ml of alkaline peptone water (Guangdong Huankai Microbial Science and Technology), after which they were incubated at 37°C for 16 h while shaking at 200 rpm. *Escherichia coli* TOP10 strains were cultured in normal LB medium under the same conditions. Genomic DNA templates were extracted using the boiled lysates method, in which 1 ml of each culture was boiled at 100°C for 8 min. After boiling, the suspension was centrifuged for 10 min at 12,000 rpm. The supernatant was then used as the DNA template. Plasmid DNA templates were extracted using a SanPrep Plasmid MiniPrep Kit (Sangon Biotech Co. Ltd.). K-serogroups of all *V. parahaemolyticus* strains were identified using commercial antisera based on agglutination tests (Denka Seiken, Tokyo, Japan) according to the manufacturer’s protocol and the Chinese National Food Safety Standards: Food Microbiological Examination *Vibrio parahaemolyticus* Testing, GB 4789.7-2013.

### Primer and Probe Design

In this study, the primers were designed as previously described (Li et al., 2019). Primers designed for development of the MLMA assay included two parts (**Table 2**). (1) The whole genome sequences of 418 *V. parahaemolyticus* strains were sequenced in the previous study (Li et al., 2019; Bian et al., 2021) and 39 pairs of primers of different K-serogroups were designed based on specific serogroup antigen genes sequences of the 418 genomes which the bioproject numbers could be obtained from the GenBank database (https://www.ncbi.nlm.nih.gov/bioproject/PRJNA677930). Among 39 pairs of primers, the eight pairs of primers were previously reported (Li et al., 2019). (2) Additionally, 18 pairs of primers targeting 18 rare K-serogroup gene sequences were designed based on the *wzy* or *wzx* gene as previously described (Pang et al., 2019) using sequence information obtained from the GenBank database (https://www.ncbi.nlm.nih.gov/). The left hybridization-ligation oligonucleotide probe (L) was purified using polyacrylamide gel electrophoresis, while the right hybridization-ligation oligonucleotide probe (R) was labeled at the 5′ end with the phosphate group (5′P) and purified using high-performance liquid chromatography. Universal primer and fluorophore-labeled probes with the reporter fluorophores carboxy-X-rhodamine (ROX), carboxyfluorescein (FAM), or indodicarbocyanine-5 (Cy5) at the 5′ end and with Black Hole Quencher at the 3′ end were obtained as described in our previous study (Jiang et al., 2017) (**Table S4**). All primers and probes were synthesized by Sangon Biotech Co. Ltd.

**Table 2:** Multiplex ligation reaction based on probe melting curve analysis (MLMA) assay for the identification of *V. parahaemolyticus* K- serogroups in a three-tube system.

Serogroup^a^	Gene loci	Tube	Fluorescence channel (Tm/◦C)	Sequence (5′-3′)	Hybridization-ligation oligonucleotide probe sequence (5′→ 3′)^b^
K3	*VP24500037*	1	(FAM,74.5)	F	**GTGGCAGGGCGCTACGAACAAT**CCTATCGGTCCTTCATCGCTCAGCCTTCACCGGTTCTTGCTGACGATCTATGTGTTAACGTAG
				R	ATGGTGATGGCGTTCTTCAGCAGATGGTGA**TGAGATTGGATCTTGCTGGGC**
K5	*VP19500014*	1	(Cy5,61.0)	F	**GTGGCAGGGCGCTACGAACAAT**CCTACGGTGAGGACCTTTGCAGATTGGCATCACCCCGGAAGGCTGTCGCTCTACTTCTATATCG
				R	AGATGAGCCAACATTTATTAGTTCGGGTT**TGAGATTGGATCTTGCTGGGC**
K6	*VP0223*	1	(ROX,74.5)	F	**GTGGCAGGGCGCTACGAACAAT**CCTAACGACTCTGGCTGCTCGTTCGTGACGCCGTTAGAACCTAAGTCTAATTATGCAGTCA
				R	CTGGGCTATATTTCTATGACAGTCGCGTAATAG**TGAGATTGGATCTTGCTGGGC**
K8	*VPBB0234*	1	(ROX,70.0)	F	**GTGGCAGGGCGCTACGAACAAT**CCTAACGACTCTAGCTGCTCGTTCGTGACGGAACTTGATTGAAGCAAGGGAACATTCTTT
				R	CGGTGAGTATGATTTAATACATTGTCACTTC**TGAGATTGGATCTTGCTGGGC**
K9	*VP13500017*	1	(FAM,66.0)	F	**GTGGCAGGGCGCTACGAACAAT**CCTATCGGTCCTTTATCGCTCACCCTTCACCGGCGGAGTGATTATAAGGAGGAGTGCTATAATG
				R	TGGGTTCGGGAATCGGTGTCAGTGTTAA**TGAGATTGGATCTTGCTGGGC**
K11	*VP10700010*	1	(Cy5,70.5)	F	**GTGGCAGGGCGCTACGAACAAT**CCTACGGTGAAGCCCTTGGCAGGTCGGTATCACCCGGTTCGGTTAAGATAAGTACTTTGGGTAGAT
				R	GTTTCATTGCACTCGCTCTGCTTATTACG**TGAGATTGGATCTTGCTGGGC**
K12	*VP17900014*	1	(FAM,51.0)	F	**GTGGCAGGGCGCTACGAACAAT**CCTATCGCTCCTTCATAGCTCAGACTTCATCGGGAACTAGTAAGTTTATATAACCCGGCATTGC
				R	ATTGTATTGAAGAGCAACGTGATTTTCCTCAATCG**TGAGATTGGATCTTGCTGGGC**
K13	*VP9800011*	1	(FAM,61.5)	F	**GTGGCAGGGCGCTACGAACAAT**CCTATCGGTCCTTCATGGCTCAGTCTTCACCGGGACCTCTTGGATGGGATTCTAATATACACG
				R	ATAAGTTTGATAAAGTATTCACTTGGAGCACTC**TGAGATTGGATCTTGCTGGGC**
K17	*VP4300018*	1	(ROX,57.5)	F	**GTGGCAGGGCGCTACGAACAAT**CCTAACGACTCTAGCTTCTCGTTAGTGACGGCTTCTTGACCACACGTTATTGTACCAAT
				R	CTTTGCTAGGTACAAAGCCAAAAGCAGCCA**TGAGATTGGATCTTGCTGGGC**
K18	*VP19700016*	1	(Cy5,53.5)	F	**GTGGCAGGGCGCTACGAACAAT**CCTACGGTGAAGCCATTGCCAGGTGGTATACCCGAGTTGGCATTGATGCTCATCTCTCCT
				R	ATCTATTCTGTTATAAAATTGAGTGTCTACGGACG**TGAGATTGGATCTTGCTGGGC**
K25	*VP13200012*	1	(ROX,66.5)	F	**GTGGCAGGGCGCTACGAACAAT**CCTAACGACTCTGTCTTCTCGTTCGTGACGGCTTATCTAGTCGTTCTTCATTTGGTGAGAAAG
				R	CTTTCAACTCCAAAAGTATCGTGATTAGAA**TGAGATTGGATCTTGCTGGGC**
K28	*VP5300035*	1	(FAM,57.0)	F	**GTGGCAGGGCGCTACGAACAAT**CCTATCCGTTCTTTATCGCTCAGCCTTCATCGGCCATATTTTGACCCTTCAGTTAGGTATCG
				R	TTGTTTCAATCTTGCTGATGAGCTAAATGAGCGA**TGAGATTGGATCTTGCTGGGC**
K29	*VP24700016*	1	(ROX,63.5)	F	**GTGGCAGGGCGCTACGAACAAT**CCTAACGACTCTAGCTGCTTGTTCGTGACGTGATAAGTATTCTTTGATATCGAAAGTGGCGA
				R	GTGTTTACAATAAGAAGATTAAAATTGAGAG**TGAGATTGGATCTTGCTGGGC**
K36	*VP12200010*	1	(FAM,70.0)	F	**GTGGCAGGGCGCTACGAACAAT**CCTATCGGTCCTTCATCGCTCGGCCTTCACCGGATTGGCAAAAAAGGTTTGTTTCAATCGAAT
				R	CTAGCAATKTGGCTCTAAGTRGTTTGAATGTAGG**TGAGATTGGATCTTGCTGGGC**
K41	*VP23400016*	1	(Cy5,74.0)	F	**GTGGCAGGGCGCTACGAACAAT**CCTACGGTGAGGCCCTTGGCAGGTTGCTATCACCCAAACAAAGCTCTCAAGGATGCTAAGCTT
				R	GATGGTGCGAGTATTTTTATTATATCGGGTGGA**TGAGATTGGATCTTGCTGGGC**
K42	*VP400015*	1	(Cy5,56.0)	F	**GTGGCAGGGCGCTACGAACAAT**CCTACGGTGCGGACCTTTGCCGATTGGCATCACCCTGTAACTCTAAACCTAAGTCTTCATGGCTGA
				R	TGTTTTGTTTTGCGGTATTGTCACAAAACTCAG**TGAGATTGGATCTTGCTGGGC**
K44	*VP11300018*	1	(Cy5,58.5)	F	**GTGGCAGGGCGCTACGAACAAT**CCTACGGTGAGGACCTTTGCCGATTGGCATCACCCGCTTTTGCAATGATAAGTAATGCTCAGGTAA
				R	TGTATCTTTCTCTACAAGAAAATCCCTTGCTTGGA**TGAGATTGGATCTTGCTGGGC**
K56	*VP33400015*	1	(ROX,60.0)	F	**GTGGCAGGGCGCTACGAACAAT**CCTAACGACACTGGCTGCTGGTCCGTGACGTAGAACACTCAAACCGGAAGTTCATCGCAA
				R	GAATGGATGCTGATGATATTTCAGAGCCA**TGAGATTGGATCTTGCTGGGC**
K60	*VP1600020*	1	(Cy5,66.0)	F	**GTGGCAGGGCGCTACGAACAAT**CCTACGGTGAAGCCCTTCGCAGGTCGGTATCACCCCTTCTTGTTACAGCCTTTAAGAGCGGGA
				R	TTGGTGCCATTTTCATCCGGATTTGGTG**TGAGATTGGATCTTGCTGGGC**
K68	*VP16100014*	1	(ROX,54.0)	F	**GTGGCAGGGCGCTACGAACAAT**CCTAACGACTATGGCTTCTCGTTGGTGACGCGATACTAATGACTCAGATGTATGCCCAGGATT
				R	TTTACAGAAATGTGGGGCCAAGAAAGTTA**TGAGATTGGATCTTGCTGGGC**
K70	*wzy*	1	(ROX,50.5)	F	**GTGGCAGGGCGCTACGAACAAT**CCTAACGACTCTATCTGCTTGTTAGTGACGATACACAATCGTTCAACATAATAAGGCAAGC
				R	ACTAGGGATGGCAATTTGTCTTTATTCGCTGGAC**TGAGATTGGATCTTGCTGGGC**
K1	*VP30500018*	2	(ROX,51.0)	F	**GTGGCAGGGCGCTACGAACAAT**CCTAACGACTCTATCTGCTTGTTAGTGACGATGGGATTAGCTATACCTAGGCTAGCCGGT
				R	GCTCATGGAGACCCTAACTATAACTCAGT**TGAGATTGGATCTTGCTGGGC**
K4	*VP31900014*	2	(ROX,54.0)	F	**GTGGCAGGGCGCTACGAACAAT**CCTAACGACTATGGCTTCTCGTTGGTGACGTTGGTATTGGTGTTGACTACGGATATGGT
				R	GCTCCAAGAATGCAAGGTTTTCTGAGTGAAC**TGAGATTGGATCTTGCTGGGC**
K19	*VP19200012*	2	(ROX,57.0)	F	**GTGGCAGGGCGCTACGAACAAT**CCTAACGACTCTAGCTTCTCGTTAGTGACGGATATGCAAGACTTGCAAAAGCTCATCACAA
				R	AATCATCTAGGTTGATGTGGGCTCTTTGT**TGAGATTGGATCTTGCTGGGC**
K20	*VP20600013*	2	(ROX,59.0)	F	**GTGGCAGGGCGCTACGAACAAT**CCTAACGACACTGGCTGCTGGTCCGTGACGGCACTGAATACGCCTTAAAAACTCTAATAGC
				R	TTGGGCTTCAGTTGTTGCAACTATTGGTGT**TGAGATTGGATCTTGCTGGGC**
K21	*VP43900016*	2	(Cy5,61.5)	F	**GTGGCAGGGCGCTACGAACAAT**CCTACGGTGAGGACCTTTGCAGATTGGCATCACCCGTTAGCTGTGGAAGACGTGTATTGTTTGAA
				R	AACTGTAACAACGACATAATGATACTATTCGATG**TGAGATTGGATCTTGCTGGGC**
K23	*VP20200014*	2	(FAM,74.0)	F	**GTGGCAGGGCGCTACGAACAAT**CCTATCGGTCCTTCATCGCTCAGCCTTCACCGGCGAAATTCATAGCGATCTTGAGACTTTCAA
				R	TCTTGGTTGTAGGAATTTATGCCATATACCA**TGAGATTGGATCTTGCTGGGC**
K30	*VP19000009*	2	(Cy5,58.5)	F	**GTGGCAGGGCGCTACGAACAAT**CCTACGGTGAGGACCTTTGCCGATTGGCATCACCCATTCAAGTCATGGATTACTGGTCCTTGTATT
				R	TTGATCGGTGCTGGTTGGTGGCAGTATGG**TGAGATTGGATCTTGCTGGGC**
K31	*VP20500017*	2	(FAM,69.5)	F	**GTGGCAGGGCGCTACGAACAAT**CCTATCGGTCCTTCATCGCTCGGCCTTCACCGGTTGGGTATGCTTCCGTCATTTAGAACTATT
				R	CACTACGGAGATACAGCCAACTACTATGAC**TGAGATTGGATCTTGCTGGGC**
K32	*VP9900015*	2	(FAM,65.5)	F	**GTGGCAGGGCGCTACGAACAAT**CCTATCGGTCCTTTATCGCTCACCCTTCACCGGCCAATCGATGAACCAAATTAGGCAATTTGC
				R	TGCAATCGCTGTGTCACTTTTTGCCTTGCT**TGAGATTGGATCTTGCTGGGC**
K33	*VP20100010*	2	(FAM,61.0)	F	**GTGGCAGGGCGCTACGAACAAT**CCTATCGGTCCTTCATGGCTCAGTCTTCACCGGGGTATTGGATTGCAGTTAGATGCGGAGTAA
				R	CGAAGCTTCATATCACCGGGATGCAAAGA**TGAGATTGGATCTTGCTGGGC**
K34	*VP1500017*	2	(ROX,70.0)	F	**GTGGCAGGGCGCTACGAACAAT**CCTAACGACTCTAGCTGCTCGTTCGTGACGCTCACAATTGTAAGACGATTACTTTACAGCA
				R	CGGATTGTATGTTGATTACTCATTAAGTGAAA**TGAGATTGGATCTTGCTGGGC**
K37	*VP23900011*	2	(FAM,57.0)	F	**GTGGCAGGGCGCTACGAACAAT**CCTATCCGTTCTTTATCGCTCAGCCTTCATCGGAGATTCTGACCTCTATGGGAAAGGGTAT
				R	GGGTTAGGGTGGTCCTTATTTAGTGATTT**TGAGATTGGATCTTGCTGGGC**
K38	*VP20000015*	2	(FAM,52.0)	F	**GTGGCAGGGCGCTACGAACAAT**CCTATCGCTCCTTCATAGCTCAGACTTCATCGGGGTATCGCCGTTACTGGCACATACCATG
				R	ATTATTGTCCGCTATCTGTTGACTTTGGGAT**TGAGATTGGATCTTGCTGGGC**
K48	*VP22900017*	2	(Cy5,55.5)	F	**GTGGCAGGGCGCTACGAACAAT**CCTACGGTGCGGACCTTTGCCGATTGGCATCACCCCCAGTTCGTCTTTGAAACGTATTTGCCAAA
				R	CGCTCTTGTTAGGCTTCCTCTCATTAATT**TGAGATTGGATCTTGCTGGGC**
K49	*VP23800014*	2	(Cy5,50.5)	F	**GTGGCAGGGCGCTACGAACAAT**CCTACGGTGAAGCCATTGCCAGGTGGTATACCTGTTTGATTATAACCCTTATGGTGCGATTCT
				R	GGGGTTAGGTTCAATGGGGTTGTATGAA**TGAGATTGGATCTTGCTGGGC**
K55	*VP100017*	2	(ROX,62.5)	F	**GTGGCAGGGCGCTACGAACAAT**CCTAACGACTCTAGCTGCTTGTTCGTGACGGCTAATTCTCAATCAAATGGATGGGACTGG
				R	TATTCATACAAAGATTCGTTCGAGCAAATTTCT**TGAGATTGGATCTTGCTGGGC**
K63	*VP3400018*	2	(ROX,66.5)	F	**GTGGCAGGGCGCTACGAACAAT**CCTAACGACTCTGTCTTCTCGTTCGTGACGCGCTAAAGGGTGGGGAGATAACGGGATA
				R	GGAATACATCAAACTGGTGGGCTAGATAATCG**TGAGATTGGATCTTGCTGGGC**
K65	*wzy*	2	(ROX,73.5)	F	**GTGGCAGGGCGCTACGAACAAT**CCTAACGACTCTGGCTGCTCGTTCGTGACGAACAAAGATTCCTTCCGGAGATAAATATTCC
				R	ATTCCTGGTATATCTGGCGTTTCGGCTGTA**TGAGATTGGATCTTGCTGGGC**
K67	*wzy*	2	(Cy5,66.0)	F	**GTGGCAGGGCGCTACGAACAAT**CCTACGGTGAAGCCCTTCGCAGGTCGGTATCACCCTATGGACGCAGTCGCGCAATGATGTTTTGCA
				R	CTGCAATTATATTATTTATGACAGCTTCCGTTCTC**TGAGATTGGATCTTGCTGGGC**
K69	*VP3300015*	2	(Cy5,70.5)	F	**GTGGCAGGGCGCTACGAACAAT**CCTACGGTGAAGCCCTTGGCAGGTCGGTATCACCCGTAGTCCTTGCATACCCCGTGTTAATAG
				R	TATCTGCCGGATTGCTCAGGAATGGACAA**TGAGATTGGATCTTGCTGGGC**
K71	*VP3200012*	2	(Cy5,74.5)	F	**GTGGCAGGGCGCTACGAACAAT**CCTACGGTGAGGCCCTTGGCAGGTTGCTATCACCCGGAATTGAAGTATGTTTCTTTGAAGAGGGA
				R	CCATGCTTTTATCGTGCAGGACAAACTAAG**TGAGATTGGATCTTGCTGGGC**
K7	*wzy*	3	(ROX,74.0)	F	**GTGGCAGGGCGCTACGAACAAT**CCTAACGACTCTGGCTGCTCGTTCGTGACGATCTTATCGAGTCAACAGCTATGTATGCGA
				R	GACTTAGTTACTTCTACTATGGCGATGGTTTGGAG**TGAGATTGGATCTTGCTGGGC**
K15	*wzy*	3	(ROX,57.0)	F	**GTGGCAGGGCGCTACGAACAAT**CCTAACGACTCTAGCTTCTCGTTAGTGACGACAGGACATACCCAAAAGACATTGGCA
				R	CTGGCTGGACTGGAACCCAGTTCTTCATG**TGAGATTGGATCTTGCTGGGC**
K22	*wzx*	3	(ROX,54.0)	F	**GTGGCAGGGCGCTACGAACAAT**CCTAACGACTATGGCTTCTCGTTGGTGACGTTTCGACAATCCAATTTCTGAGCGAGTCTTTA
				R	CCTTCATTTATGCGTTTACAAATCCATCTAC**TGAGATTGGATCTTGCTGGGC**
K24	*wzy*	3	(ROX,70.0)	F	**GTGGCAGGGCGCTACGAACAAT**CCTAACGACTCTAGCTGCTCGTTCGTGACGCTTACACGCAATTTTAGAGGCGTTACAATAT
				R	CTGCTATTGGTATTGGGATTGCACTATCTCTAGC**TGAGATTGGATCTTGCTGGGC**
K39	*wzy*	3	(ROX,50.5)	F	**GTGGCAGGGCGCTACGAACAAT**CCTAACGACTCTATCTGCTTGTTAGTGACGGTGGTAGGTTCTTTGCATACATACTGCTCCTG
				R	AACGATTCTGTGTTTCGTACAACTTATATGGTGTA**TGAGATTGGATCTTGCTGGGC**
K40	*wzy*	3	(ROX,62.5)	F	**GTGGCAGGGCGCTACGAACAAT**CCTAACGACTCTAGCTGCTTGTTCGTGACGATGATAGCAAAGATGTCGCTCATATAACGAG
				R	TTGGGAGATGGGGTTTCAAGCATTATTTAGAGTT**TGAGATTGGATCTTGCTGGGC**
K43	*wzy*	3	(ROX,66.5)	F	**GTGGCAGGGCGCTACGAACAAT**CCTAACGACTCTGTCTTCTCGTTCGTGACGTTTGCGTTGTCTCTATTGTATTACTTTGCG
				R	CTATATTTCCTAGCTAAGGAAATGGGGCAGATAAG**TGAGATTGGATCTTGCTGGGC**
K45	*wzy*	3	(ROX,59.0)	F	**GTGGCAGGGCGCTACGAACAAT**CCTAACGACACTGGCTGCTGGTCCGTGACGAATACTCTATGGGTGGCGATATATTGCGATA
				R	GGCTATGGCGGGATTGATGCAAGTGCTTATAAG**TGAGATTGGATCTTGCTGGGC**
K46	*wzy*	3	(Cy5,58.5)	F	**GTGGCAGGGCGCTACGAACAAT**CCTACGGTGAGGACCTTTGCCGATTGGCATCACCCTGCAGCACTCTCTTGATTACAGTGATGATTA
				R	CGGTCTCGCATCAATTCTGGCTACTTTTGCTGC**TGAGATTGGATCTTGCTGGGC**
K51	*wzy*	3	(Cy5,61.5)	F	**GTGGCAGGGCGCTACGAACAAT**CCTACGGTGAGGACCTTTGCAGATTGGCATCACCCATGCATTTAAAGGAGTTACAACCACTCCA
				R	CAGGCTCTGGCCTCGCTTATTATTTTAGG**TGAGATTGGATCTTGCTGGGC**
K52	*wzx*	3	(Cy5,66.0)	F	**GTGGCAGGGCGCTACGAACAAT**CCTACGGTGAAGCCCTTCGCAGGTCGGTATCACCCAGCTATCTGATGACCTTGTGGCATTTAAA
				R	GAAATGGTTTCTAAAGTAGTTCCTATTGTCGCCTT**TGAGATTGGATCTTGCTGGGC**
K53	*wzy*	3	(Cy5,74.0)	F	**GTGGCAGGGCGCTACGAACAAT**CCTACGGTGAGGCCCTTGGCAGGTTGCTATCACCCATGGTGAATTCTGGGTAAGAAACAACCG
				R	TACGAGTCTTTGGGGGCAATATGTATTTTCAGCTC**TGAGATTGGATCTTGCTGGGC**
K54	*wzy*	3	(Cy5,53.0)	F	**GTGGCAGGGCGCTACGAACAAT**CCTACGGTGAAGCCATTGCCAGGTGGTATACCCTGTCAGACTCCGGAATTCCCGCTTTATCAT
				R	CTATAAAGACTTTGCTCTACGATTCAAGTAATTCG**TGAGATTGGATCTTGCTGGGC**
K59	*wzy*	3	(Cy5,56.0)	F	**GTGGCAGGGCGCTACGAACAAT**CCTACGGTGCGGACCTTTGCCGATTGGCATCACCCACGACGAGTGTTCAATTGGATGACGATAGTTC
				R	AATGTTTATCTCTCTACTAAACGGAGAGTTTAGGAG**TGAGATTGGATCTTGCTGGGC**
K64	*wzy*	3	(Cy5,70.5)	F	**GTGGCAGGGCGCTACGAACAAT**CCTACGGTGAAGCCCTTGGCAGGTCGGTATCACCCAAGCCTTTCAGCCATCCCCAAGAATCCA
				R	CCTGTTCTTGGAGAGTTAAGACCATCAATACT**TGAGATTGGATCTTGCTGGGC**
IC	*SUC2*	3	(FAM,65.0)	F	**GTGGCAGGGCGCTACGAACAAT**CCTATCGGTCCTTTATCGCTCACCCTTCACCGGGATCGCATGACTCAGTCATCGTGAAA
				R	GAAAGGCACAACTTTGTAGAGATTTCTGT**TGAGATTGGATCTTGCTGGGC**
VP	*toxR*	3	(FAM,70.0)	F	**GTGGCAGGGCGCTACGAACAAT**CCTATCGGTCCTTCATCGCTCGGCCTTCACCGGAACCAGAAGCGCCAGTAGTACCTGAAAAAGCA
				R	CCTGTGGCTTCTGCTGTGAATCCTTGGATT**TGAGATTGGATCTTGCTGGGC**

^a^K3, K6, K8, K9, K25, K29, K56 and K68 were the 8 previously reported K-serogroups; K7, K15, K22, K24, K39, K40, K43, K45, K46, K51, K52, K53, K54, K59, K64, K65, K67 and K70 were the 18 rare K-serogroups.

^b^Bold typeface indicates universal primer sequences used during the LATE-PCR amplification step; whereas fluorescent detection probe sequences are underlined.

### Design of the MLMA Assay

The two key steps involved in the MLMA assay are (1) a hybridization-ligation process and (2) a PCR amplification and melt curve analysis (Li et al., 2019). In step 1, the reaction is conducted in a 10 µl reaction mixture containing 1.5 µl of ligation probe mix (0.63–10.0 nM of each hybridization oligonucleotide probe) (**Table 2**), 1 U Taq DNA ligase and 1 µl DNA ligase buffer (New England Biolabs, Beijing, China), 1.5 µl sterilized water, and 5 µl DNA template. The reaction was performed in a T3 Thermocycler (Biometra, Germany) under the following parameters: denaturation of the initial reaction, which only contained ligation probe mix and DNA template, at 95°C for 5 min, followed by a reduction to 75°C to pause the reaction, after which 3.5 µl containing the remainder of the reaction mixture were added and the samples were incubated at 60°C for 60 min, followed by 95°C for 5 min. In step 2, the reaction was conducted in a 50 µl reaction mixture containing 1×PCR buffer, 1.5 mM MgCl2, 0.2 mM deoxynucleoside triphosphate, 1 U Taq polymerase, 0.015 µM limiting primer, 0.3 µM excess primer, 0.12 µM fluorogenic probes, and 5 µl of ligation product from step 1. The reaction was performed in a BioRad CFX96 real-time PCR system (BioRad Inc., Hercules, CA, USA) under the following conditions: host start at 95°C for 3 min, then 38 cycles of 95°C for 10 s, 57°C for 20 s, and 72°C for 20 s, followed by 95°C for 1 min, 40°C for 2 min, and then an increase to 85°C in 1°C steps with 5 s between each step. All fluorescent signal intensity was captured by ROX, FAM, and Cy5 channels. The melting temperature (Tm) values of melt curve analysis were obtained automatically using CFX Manager 3.0 software.

### Analytical Performance of the MLMA Assay

To determine the limit of detection (LOD) of the assay, a series of 10-fold dilutions in triplicates of purified DNA from 10.0 ng/µl to 0.01 ng/µl were analyzed. To evaluate the intra-assay and inter-assay reproducibility, two sets of 10-fold dilutions in triplicate from 10.0 ng/µl to 1.0 ng/µl were analyzed and the standard deviations and coefficient of variation values were calculated. Each concentration of the assay was analyzed in triplicate.

### Evaluation of the MLMA Assay Using a Double-Blind Test

The 595 strains were selected to evaluate the specificity and sensitivity of the MLMA assay. The K-serogroups of the 595 isolates were distinguished based on the MLMA assay method compared with the agglutination tests (Denka Seiken) using commercial antisera according to the manufacturer’s instructions with a double-blind test. The genomic DNA was extracted from the 595 isolates using the boiled lysates protocol. Procedures were performed according to the protocols described above. Evaluation of consistency between the MLMA assay and traditional serological methods was performed using Kappa analysis.

### Statistical Analysis

The SPSS 23.0 statistical software package was used for statistical analysis. The positive detection rate of the MLMA assay and traditional serological method was compared with the paired χ^2^ test, and a P<0.01 was considered to indicate statistical significance.

### Ethics

*V. parahaemolyticus* isolates from the Shenzhen Center for Disease Control and Prevention were de-identified and anonymized to protect patient privacy and confidentiality. Therefore, ethical clearance was not required.

## Results

### Identification of 57 *V. parahaemolyticus* K-Serogroups

A three-tube system was set up with three fluorescence channels (ROX, FAM, and Cy5) in each tube. In the first tube, the ROX channel detected the K-serogroups K70, K68, K17, K56, K29, K25, K8, and K6, the Cy5 channel detected the K-serogroups K18, K42, K44, K5, K60, K11, and K41 and the FAM channel detected the K-serogroups K12, K28, K13, K9, K36, and K3. In the second tube, the ROX channel detected the K-serogroups K1, K4, K19, K20, K55, K63, K34, and K65, the Cy5 channel detected K49, K48, K30, K21, K67, K69, and K71 and the FAM channel detected the K-serogroups K38, K37, K33, K32, K31, and K23. In the third tube, the ROX channel detected the K-serogroups K39, K22, K15, K45, K40, K43, K24, and K7, the Cy5 channel detected the K-serogroups K54, K59, K46, K51, K52, K64, and K53 and the FAM channel detected the *SUC2* gene as an internal control (IC) and the *toxR* gene as a confirmation of *V. parahaemolyticus*. The combined use of fluorescence channels and designed Tm values for targeting specific gene loci of K-serogroups in each tube are listed in **Table 3**.

**Table 3:** Identification matrix of gene targets in a three-tube multiplex ligation reaction based on probe melting curve analysis (MLMA) system.

First tube	Antigen	Tm(°C)	Second tube	Antigen	Tm(°C)	Third tube	Antigen	Tm(°C)
ROX channel	K70	50.5	ROX channel	K1	51.0	ROX channel	K39	50.5
	K68	54.0		K4	54.0		K22	54.0
	K17	57.5		K19	57.0		K15	57.0
	K56	60.0		K20	59.0		K45	59.0
	K29	63.5		K55	62.5		K40	62.5
	K25	66.5		K63	66.5		K43	66.5
	K8	70.0		K34	70.0		K24	70.0
	K6	74.5		K65	73.5		K7	74.0
Cy5 channel	K18	53.5	Cy5 channel	K49	50.5	Cy5 channel	K54	53.0
	K42	56.0		K48	55.5		K59	56.0
	K44	58.5		K30	58.5		K46	58.5
	K5	61.0		K21	61.5		K51	61.5
	K60	66.0		K67	66.0		K52	66.0
	K11	70.5		K69	70.5		K64	70.5
	K41	74.0		K71	74.5		K53	74.0
FAM channel	K12	51.0	FAM channel	K38	52.0	FAM channel	IC	65.0
	K28	57.0		K37	57.0		VP	70.0
	K13	61.5		K33	61.0			
	K9	66.0		K32	65.5			
	K36	70.0		K31	69.5			
	K3	74.5		K23	74.0			

### Performance Characteristics of the MLMA Assay

**Figure 1:**
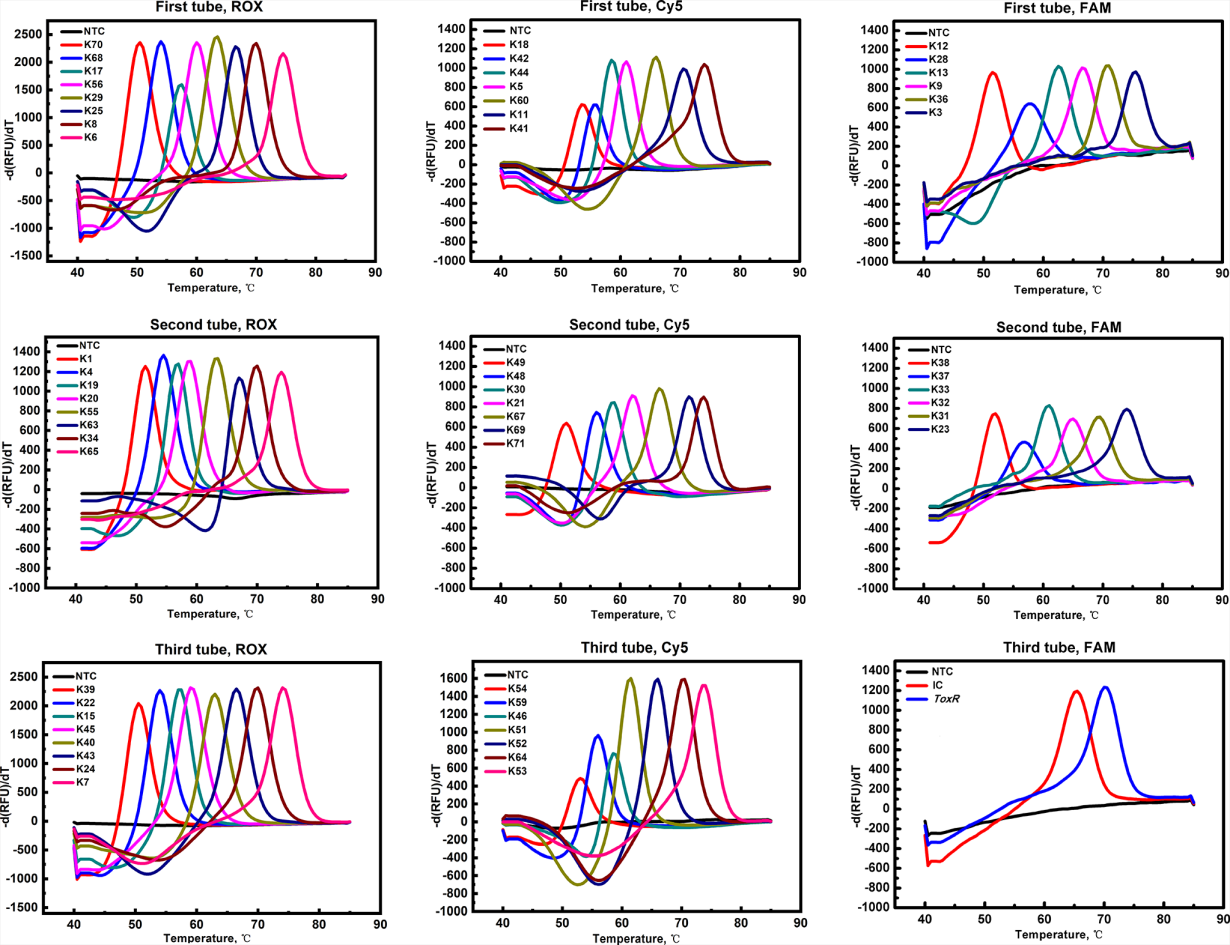
Probe melting curve analysis for the identification of *V. parahaemolyticus*. *57* K-serogroups in the multiplex ligation reaction based on probe melting curve analysis (MLMA) assay. Color-coded melting curves represent the different antigen genes in each fluorophore channel (ROX, FAM, and Cy5) in a three-tube system. IC, SUC2 gene was used as a positive internal control (IC); NTC, negative control.

With the use of 59 pairs of ligation oligonucleotides in a three-tube system, the MLMA assay was able to detect all target genes of 57 K-serogroups and one IC and the *toxR* gene, which yielded expected Tm values that represented distinguished K-serogroups (**Figure 1**). The specificity study showed no cross-reactivity among the 57 K-serogroups. The limit of detection (LOD) of the assay for all target genes of K-serogroups ranged from 0.1 to 1.0 ng/µl at the DNA level. The intra-assay and inter-assay reproducibility study demonstrated that the measurement of Tm values has highly reproducible traits. Specifically, the largest SD value of the mean Tm was no more than 1°C and the CV value ranged from 0 to 1% (**Table 4**).

**Table 4:** Reproducibility of designated melting temperatures (Tm) and Limit of Identification values for each serogroup target loci in the assay.

Name	Gene	Tube	Concentration	Intra-assay reproducibility			Inter-assay reproducibility			Limit of identification
			ng/µl	Mean Tm (°C)	SD	CV (%)	Mean Tm (°C)	SD	CV (%)	(ng/µl)
K3	*VP24500037*	1	10.0	74.50	0.00	0.00	74.78	0.53	0.71	0.10
			1.0	74.50	0.00	0.00	74.61	0.31	0.42	
K5	*VP19500014*	1	10.0	61.00	0.00	0.00	61.00	0.24	0.39	0.10
			1.0	61.00	0.00	0.00	61.00	0.00	0.00	
K6	*VP0223*	1	10.0	74.50	0.00	0.00	74.44	0.16	0.21	0.10
			1.0	74.17	0.24	0.32	74.22	0.25	0.33	
K8	*VPBB0234*	1	10.0	70.00	0.00	0.00	69.78	0.25	0.36	0.10
			1.0	69.50	0.00	0.00	69.50	0.24	0.34	
K9	*VP13500017*	1	10.0	65.83	0.24	0.36	65.61	0.31	0.48	0.10
			1.0	65.50	0.41	0.62	65.39	0.46	0.70	
K11	*VP10700010*	1	10.0	70.33	0.24	0.34	70.33	0.24	0.34	0.10
			1.0	70.50	0.00	0.00	70.44	0.16	0.22	
K12	*VP17900014*	1	10.0	50.83	0.24	0.46	50.83	0.24	0.46	1.00
			1.0	50.67	0.24	0.47	50.78	0.25	0.49	
K13	*VP9800011*	1	10.0	61.33	0.24	0.38	61.67	0.47	0.76	0.10
			1.0	61.17	0.24	0.39	61.17	0.24	0.39	
K17	*VP4300018*	1	10.0	57.50	0.00	0.00	57.39	0.21	0.36	0.10
			1.0	57.17	0.24	0.41	57.17	0.24	0.41	
K18	*VP19700016*	1	10.0	53.50	0.00	0.00	53.61	0.21	0.39	0.10
			1.0	53.83	0.24	0.44	53.72	0.25	0.46	
K25	*VP13200012*	1	10.0	66.50	0.00	0.00	66.56	0.16	0.24	1.00
			1.0	66.33	0.24	0.36	66.28	0.25	0.37	
K28	*VP5300035*	1	10.0	57.00	0.00	0.00	57.33	0.47	0.82	0.10
			1.0	56.83	0.24	0.41	56.83	0.24	0.41	
K29	*VP24700016*	1	10.0	63.33	0.24	0.37	63.39	0.21	0.33	0.10
			1.0	63.00	0.00	0.00	63.06	0.16	0.25	
K36	*VP12200010*	1	10.0	69.83	0.24	0.34	69.78	0.34	0.49	0.10
			1.0	69.83	0.24	0.34	69.83	0.33	0.48	
K41	*VP23400016*	1	10.0	74.17	0.24	0.32	74.00	0.24	0.32	0.10
			1.0	74.17	0.24	0.32	74.11	0.21	0.28	
K42	*VP400015*	1	10.0	55.83	0.24	0.42	55.94	0.16	0.28	1.00
			1.0	56.00	0.00	0.00	56.00	0.00	0.00	
K44	*VP11300018*	1	10.0	58.50	0.00	0.00	58.50	0.00	0.00	0.10
			1.0	58.50	0.00	0.00	58.50	0.00	0.00	
K56	*VP33400015*	1	10.0	60.00	0.00	0.00	59.94	0.16	0.26	0.10
			1.0	59.50	0.00	0.00	59.56	0.16	0.26	
K60	*VP1600020*	1	10.0	66.00	0.00	0.00	65.94	0.16	0.24	0.10
			1.0	66.00	0.00	0.00	66.06	0.16	0.24	
K68	*VP16100014*	1	10.0	54.00	0.00	0.00	54.06	0.16	0.29	0.10
			1.0	54.00	0.00	0.00	53.94	0.16	0.29	
K70	*wzy*	1	10.0	50.50	0.00	0.00	50.44	0.16	0.31	0.10
			1.0	50.33	0.24	0.47	50.39	0.21	0.41	
K1	*VP30500018*	2	10.0	50.83	0.29	0.57	50.83	0.25	0.49	0.10
			1.0	50.33	0.29	0.57	50.67	0.35	0.70	
K4	*VP31900014*	2	10.0	54.00	0.00	0.00	54.00	0.00	0.00	0.10
			1.0	53.50	0.00	0.00	53.72	0.26	0.49	
K19	*VP19200012*	2	10.0	57.00	0.00	0.00	56.89	0.22	0.39	0.10
			1.0	56.33	0.29	0.51	56.50	0.35	0.63	
K20	*VP20600013*	2	10.0	59.00	0.00	0.00	59.00	0.00	0.00	0.10
			1.0	58.67	0.29	0.49	58.67	0.25	0.43	
K21	*VP43900016*	2	10.0	62.00	0.00	0.00	61.83	0.25	0.40	1.00
			1.0	61.00	0.00	0.00	61.17	0.25	0.41	
K23	*VP20200014*	2	10.0	74.00	0.00	0.00	74.17	0.25	0.34	0.10
			1.0	74.00	0.00	0.00	74.28	0.36	0.49	
K30	*VP19000009*	2	10.0	58.67	0.29	0.49	58.61	0.22	0.38	0.10
			1.0	58.50	0.00	0.00	58.50	0.00	0.00	
K31	*VP20500017*	2	10.0	69.50	0.00	0.00	69.67	0.25	0.36	1.00
			1.0	69.50	0.00	0.00	69.72	0.26	0.38	
K32	*VP9900015*	2	10.0	65.50	0.00	0.00	65.72	0.26	0.40	0.10
			1.0	65.67	0.29	0.44	65.83	0.35	0.54	
K33	*VP20100010*	2	10.0	61.00	0.00	0.00	61.11	0.33	0.55	0.10
			1.0	61.17	0.29	0.47	61.17	0.35	0.58	
K34	*VP1500017*	2	10.0	69.83	0.29	0.41	69.94	0.17	0.24	0.10
			1.0	70.00	0.00	0.00	69.89	0.22	0.32	
K37	*VP23900011*	2	10.0	57.00	0.00	0.00	57.17	0.25	0.44	1.00
			1.0	56.67	0.29	0.51	56.72	0.26	0.46	
K38	*VP20000015*	2	10.0	51.67	0.29	0.56	51.83	0.25	0.48	0.10
			1.0	51.50	0.00	0.00	51.56	0.30	0.58	
K48	*VP22900017*	2	10.0	55.83	0.29	0.52	55.61	0.22	0.40	0.10
			1.0	55.50	0.00	0.00	55.61	0.22	0.40	
K49	*VP23800014*	2	10.0	50.50	0.00	0.00	50.44	0.17	0.33	0.10
			1.0	50.17	0.29	0.58	50.33	0.25	0.50	
K55	*VP100017*	2	10.0	62.50	0.00	0.00	62.67	0.25	0.40	0.10
			1.0	62.50	0.00	0.00	62.67	0.25	0.40	
K63	*VP3400018*	2	10.0	66.50	0.00	0.00	66.50	0.00	0.00	0.10
			1.0	66.33	0.29	0.44	66.44	0.17	0.25	
K65	*wzy*	2	10.0	73.50	0.00	0.00	73.67	0.25	0.34	0.10
			1.0	73.50	0.00	0.00	73.72	0.26	0.36	
K67	*wzy*	2	10.0	66.50	0.00	0.00	66.17	0.25	0.38	0.10
			1.0	65.67	0.29	0.44	65.72	0.26	0.40	
K69	*VP3300015*	2	10.0	71.50	0.00	0.00	71.00	0.43	0.61	0.10
			1.0	70.33	0.29	0.41	70.50	0.25	0.35	
K71	*VP3200012*	2	10.0	74.50	0.00	0.00	74.56	0.17	0.22	0.10
			1.0	73.67	0.29	0.39	73.67	0.25	0.34	
K7	*wzy*	3	10.0	74.00	0.00	0.00	73.94	0.16	0.21	0.10
			1.0	73.83	0.24	0.32	73.78	0.25	0.34	
K15	*wzy*	3	10.0	57.17	0.24	0.41	57.11	0.21	0.36	0.10
			1.0	57.17	0.24	0.41	57.11	0.21	0.36	
K22	*wzx*	3	10.0	54.00	0.00	0.00	54.00	0.00	0.00	0.10
			1.0	53.83	0.24	0.44	53.78	0.25	0.46	
K24	*wzy*	3	10.0	70.00	0.00	0.00	70.00	0.00	0.00	0.10
			1.0	70.00	0.41	0.58	70.00	0.33	0.48	
K39	*wzy*	3	10.0	50.50	0.00	0.00	50.56	0.16	0.31	0.10
			1.0	50.33	0.24	0.47	50.39	0.39	0.78	
K40	*wzy*	3	10.0	62.50	0.00	0.00	62.72	0.25	0.40	0.10
			1.0	62.67	0.24	0.38	62.83	0.33	0.53	
K43	*wzy*	3	10.0	66.50	0.00	0.00	66.50	0.00	0.00	0.10
			1.0	66.33	0.24	0.36	66.39	0.21	0.31	
K45	*wzy*	3	10.0	59.00	0.00	0.00	59.06	0.16	0.27	0.10
			1.0	59.17	0.24	0.40	59.06	0.28	0.48	
K46	*wzy*	3	10.0	58.67	0.24	0.40	58.67	0.24	0.40	0.10
			1.0	58.67	0.24	0.40	58.61	0.31	0.54	
K51	*wzy*	3	10.0	61.50	0.00	0.00	61.44	0.16	0.26	0.10
			1.0	61.33	0.24	0.38	61.28	0.25	0.41	
K52	*wzx*	3	10.0	66.00	0.00	0.00	65.94	0.16	0.24	0.10
			1.0	65.83	0.24	0.36	65.72	0.34	0.52	
K53	*wzy*	3	10.0	74.00	0.00	0.00	73.78	0.25	0.34	0.10
			1.0	73.83	0.24	0.32	73.78	0.25	0.34	
K54	*wzy*	3	10.0	53.00	0.00	0.00	53.00	0.00	0.00	0.10
			1.0	53.00	0.00	0.00	53.00	0.00	0.00	
K59	*wzy*	3	10.0	56.00	0.00	0.00	56.00	0.00	0.00	0.10
			1.0	56.00	0.00	0.00	56.00	0.00	0.00	
K64	*wzy*	3	10.0	70.50	0.00	0.00	70.39	0.21	0.30	0.10
			1.0	70.33	0.24	0.34	70.33	0.24	0.34	
IC	*SUC2*	3	10.0	65.50	0.00	0.00	65.06	0.37	0.57	0.10
			1.0	65.17	0.47	0.72	64.94	0.37	0.57	
VP	*toxR*	3	10.0	70.17	0.24	0.34	69.72	0.48	0.69	0.10
			1.0	69.83	0.24	0.34	69.67	0.47	0.68	

### Evaluation of the MLMA Assay

Of the 595 isolates, 377 were identified and classified to 29 K-serogroups, and the remaining 218 isolates were untypeable by using the MLMA assay. Among 377 isolates, the 18 could be serotyped using the MLMA assay, but could not be serotyped using the conventional serological method. Sanger sequencing of the 18 isolates demonstrated 100% concordance with the MLMA assay (**Table S5**). Evaluation using the McNemar test revealed that the difference was statistically significant (P=0.00), the positive detection rate of MLMA (63.36%) was higher than that of conventional serotyping (60.34%), and the MLMA assay sensitivity and specificity were 100% and 92.37%, respectively. Moreover, the MLMA assay showed 96.97% consistency with the conventional serological method for all isolates. The kappa value between the MLMA assay and the traditional serological method was 0.936 (**Table 5**). The results of identification using the MLMA assay and the traditional serological method are listed in **Table S6**. In addition, five rare K-serogroups, K24, K52, K54, K67, and K70, were identified using the established MLMA assay (**Table S7**).

**Table 5:** The results of serotype identification by multiplex ligation reaction based on probe melting curve analysis (MLMA) assay and conventional serotyping tests among *V. parahaemolyticus* isolates (n = 595) from the double-blind study.

MLMA	Conventional serotyping		Total	Sensitivity	Specificity	Consistency rate	Kappa value
	+	−					
+	359	18	377				
−	0	218	218	100%	92.37%	96.97%	0.936 (P = 0.00)
**Total**	359	236	595				

MLMA (+) and Conventional serotyping (+）indicates that the results of both tests are of the same specific K-serogroups.

MLMA (+) and Conventional serotyping (−）indicates that the former results are specific K-serogroups and the latter are KUT.

MLMA (−) and Conventional serotyping (−) indicates that the results of both tests are KUT.

MLMA (−) and Conventional serotyping (+）indicates that the former results are KUT and the latter are specific K-serogroups.

## Discussion

*V. parahaemolyticus* is the leading causal agent of human acute gastroenteritis and has become a global public health issue (Letchumanan et al., 2014; Han et al., 2019). *V. parahaemolyticus* serotyping is considered to be the initial step for timely detection and source tracking of vibriosis outbreaks and can provide historical and comparable data for the surveillance of *V. parahaemolyticus* infections on a global scale (Han et al., 2016; Baker-Austin et al., 2018). Thus, the development of rapid and robust serotyping methods can facilitate national and international surveillance of *V. parahaemolyticus* infections.

In this study, we developed a molecular assay for the simultaneous identification of 57 *V. parahaemolyticus* K-serogroups based on the principles of MLMA. The assay can detect all gene targets with expected Tm values and no cross-reaction was observed. The detection limit of the assay ranged from 0.1 to 1.0 ng/µl. Moreover, the intra-assay and inter-assay reproducibility analyses showed that the assay had highly reproducible traits, with the largest SD value for the mean Tm being no more than 1°C and the CVs being less than 1%. Overall, the MLMA assay demonstrated a 96.97% consistency rate with the conventional serological method of all isolates and a kappa value of 0.936, which indicated the consistency of the two approaches is excellent.

With the growing number of genomic sequences and serogroup-specific gene sequences available in public databases, various molecular serotyping methods have been developed as alternatives for serotyping, such as common and real-time PCR, multiplex PCR-based microarrays, Luminex-based serotyping assays and sequencing-based approaches (Liu et al., 2015; Cabral-Castro et al., 2016; Afroj et al., 2017; Pang et al., 2019). Although crucial progress regarding antigen synthesis gene clusters and serogroup-specific genes for *Salmonella* and *Escherichia coli*, there has been little progress on the capsular polysaccharide gene clusters or genetic targets of *V. parahaemolyticus* K-antigens to date. Thus, the development of a molecular method for *V. parahaemolyticus* K-serogroups is highly dependent on the investigation of the genetic characteristics of *V. parahaemolyticus* K-antigen. Early studies of K-antigen genetic determinants in *V. parahaemolyticus* produced controversial results (Guvener and Mccarter, 2003 Okura et al., 2008). Later, in 2010, the detailed genetic determinants of K-antigen synthesis were confirmed to be between *gmhD* and *rjg* using an O3:K6 isolate based on the construction of gene deletions (Chen et al., 2010). We used the whole genome sequence of 418 *V. parahaemolyticus* strains to design the target genes primers and probes and then analyzed the genetic structure and evolutionary relationship of their 39 K-serogroups to identify the serogroup-specific genes of the capsular polysaccharide gene clusters (Bian et al., 2021). Additionally, another capsular polysaccharide gene clusters of 18 rare K-serogroups that we lack these data were analyzed (Pang et al., 2019). Thus, 57 K-serogroups specific sequences were selected for the development of *V. parahaemolyticus* serotyping assay.

Currently, only three assays have the ability to detect more than two *V. parahaemolyticus* K-serogroups, the first is a microsphere-based suspension array, the second is the sequence-based serotyping of *V. parahaemolyticus* 55 K-serogroups, and the third is the MLMA assay we presented for the simultaneous identification of nine K-serogroups (Li et al., 2019; Pang et al., 2019). However, the microsphere-based suspension array and sequence-based serotyping method require expensive equipment with related software and rely on bioinformatics analysis. In this study, we expanded the high throughput targets of our previously developed MLMA assay from nine K-serogroups to 57 K-serogroups and identified five rare K-serogroups using the method. To date, we have been able to detect 12 O-serogroups (Li et al., 2019) and 57 K-serogroups in four tubes using the established MLMA assay.

Upon the evaluation of the assay using 595 isolates over 15 years (2003–2018) from the Shenzhen Center for Disease Control and Prevention, the MLMA assay accurately detected 377 typeable isolates that belonged to 29 K-serogroups and 218 untypeable isolates. In addition, there were inconsistent results for 18 isolates generated by the MLMA assay and the serological method. The results of Sanger sequencing of the PCR amplicons of these isolates were consistent with the results of the MLMA assay, which indicates the MLMA assay has higher sensitivity and accuracy than the serological method. Among 377 typeable isolates, five rare K-serogroups were detected (2008–2017) by the MLMA assay, which suggested that *V. parahaemolyticus* with extremely rare K-serogroups emerged in Shenzhen as early as 2008, and that routine monitoring should be conducted. The current assay showed similar superior performances of the assay as previously reported. However, the entire MLMA assay could be completed within 3.5–4.0 h, which saves 12–18 h when compared with traditional serotyping (Li et al., 2019). Additionally, a simple boiled lysates protocol is used for the preparation of DNA templates, which also increases the flexibility of the MLMA assay. As demonstrated by the analytical studies, the MLMA assay could accurately and reproducibly detect 57 *V. parahaemolyticus* K-serogroups. Hence, the targets of the assay covered approximately 81% *V. parahaemolyticus* K-serogroups that have been identified worldwide, demonstrating that multiplex assays facilitate timely and effective detection.

However, this assay was unable to differentiate two closely related K-serogroup pairs, K56 and K57, K47, and K48, which could be attributed to their capsular polysaccharide gene clusters showing almost identical homology to the corresponding regions (Pang et al., 2019). Currently, genetic markers for the accurate identification of these two pairs are unavailable and some strains were still untypeable. Therefore, further research is necessary to enable differentiation of these serogroups at the molecular level. Furthermore, with the development of whole-genome sequencing, the increasing demand for the sequence-based serotyping method gives us an important consideration to combine the MLMA assay with the genotyping for more effective foodborne disease outbreak investigation, source tracing, and the surveillance of *V. parahaemolyticus* infection.

In a word, a novel molecular assay was developed for simultaneous detection of 57 K-serogroups of *V. parahaemolyticus* and five rare K-serogroups were found. This assay can provide a rapid, accurate, and highly sensitive identification of *V. parahaemolyticus* K-serogroups and is valuable for screening large samples during vibriosis outbreaks and surveillance.

## Data Availability Statement

The datasets presented in this study can be found in online repositories. The names of the repository/repositories and accession number(s) can be found in the article/**Supplementary Material** (**Table S8** and **Table S9**).

## Author Contributions

LLu and ML conceived and designed the experiments. LLu and ML performed the experiments and contributed to analysis. LLu, ML, and QH wrote the paper. YLi, MJ, and XS guided the selection of experimental strains. YQ and RC checked the information of strains. YJ, LZ, and LW guided the experimental operation. YL and QL provided experimental technical support. SB and LLi analyzed the gene sequence. LLu and ML contributed equally to this article. All authors contributed to the article and approved the submitted version.

## Funding

This research was supported by the China National Science and Technology Major Projects Foundation (No. 2017ZX10303406), National Natural Science Foundation of China (No. 81773436), Sanming Project of Medicine in Shenzhen (No. SZSM201811071), Shenzhen Key Medical Discipline Construction Fund (SZXK064), and the Shenzhen Health and Family Planning Commission (SZGW2017004).

## Conflict of Interest

SB and LLi was employed by BGI-Shenzhen.

The remaining authors declare that the research was conducted in the absence of any commercial or financial relationships that could be construed as a potential conflict of interest.
