# Supplementary material for: A Novel Molecular Method for Simultaneous Identification of Vibrio parahaemolyticus 57 K-Serogroups Using Probe Melting Curve Analysis
Source: Front Cell Infect Microbiol. 2021 Feb 26;11:594808. doi: 10.3389/fcimb.2021.594808 (PMC7953158; doi:10.3389/fcimb.2021.594808)
Supplement: Supplementary file 1 [file Table_1.docx]

| **TABLE S1 \|** Rare *V.parahaemolyticus* K-serogroups isolates (n=18) using *Escherichia coli* TOP10 strains. | |
| --- | --- |
| **Serotype** | **Number of isolates** |
| K7 | 1 |
| K15 | 1 |
| K22 | 1 |
| K24 | 1 |
| K39 | 1 |
| K40 | 1 |
| K43 | 1 |
| K45 | 1 |
| K46 | 1 |
| K51 | 1 |
| K52 | 1 |
| K53 | 1 |
| K54 | 1 |
| K59 | 1 |
| K64 | 1 |
| K65 | 1 |
| K67 | 1 |
| K70 | 1 |

| **TABLE S2 \|** The general information for the 359 *V. parahaemolyticus* strains. | | | | | |
| --- | --- | --- | --- | --- | --- |
| **No.** | **Sample ID*** | **Serotype** | **Year** | **Source** | **Location** |
| 1 | VP03040 | O1:K25 | 2003 | clinical | Shenzhen Center for Disease Control and Prevention |
| 2 | VP04102 | O1:K25 | 2004 | clinical | Nanshan District Center for Disease Control and Prevention |
| 3 | VP04108 | OUT:K6 | 2004 | clinical | Nanshan District Center for Disease Control and Prevention |
| 4 | VP04126 | O3:K68 | 2004 | clinical | Nanshan District Center for Disease Control and Prevention |
| 5 | VP04129 | O1:K6 | 2004 | clinical | Nanshan District Center for Disease Control and Prevention |
| 6 | VP04137 | OUT:K8 | 2004 | clinical | Nanshan District Center for Disease Control and Prevention |
| 7 | VP04138 | O6:K8 | 2004 | clinical | Nanshan District Center for Disease Control and Prevention |
| 8 | VP05238 | OUT:K8 | 2005 | clinical | Luohu District Center for Disease Control and Prevention |
| 9 | VP05272 | O1:K8 | 2005 | clinical | Shenzhen Center for Disease Control and Prevention |
| 10 | VP05286 | OUT:K42 | 2005 | food | Shenzhen Center for Disease Control and Prevention |
| 11 | VP06021 | O1:K25 | 2006 | clinical | Nanshan District Center for Disease Control and Prevention |
| 12 | VP06061 | O1:K6 | 2006 | clinical | Yantian District Center for Disease Control and Prevention |
| 13 | VP06073 | O4:K6 | 2006 | food | Luohu District Center for Disease Control and Prevention |
| 14 | VP06083 | OUT:K9 | 2006 | clinical | Futian District Center for Disease Control and Prevention |
| 15 | VP06089 | OUT:K6 | 2006 | clinical | Futian District Center for Disease Control and Prevention |
| 16 | VP06109 | O1:K6 | 2006 | clinical | Luohu District Center for Disease Control and Prevention |
| 17 | VP06190 | O6:K18 | 2006 | clinical | Luohu District Center for Disease Control and Prevention |
| 18 | **VP07001** | O4:K8 | 2007 | food | Luohu District Center for Disease Control and Prevention |
| 19 | **VP07015** | O1:K25 | 2007 | clinical | Futian District Center for Disease Control and Prevention |
| 20 | **VP07023** | O3:K6 | 2007 | clinical | Shenzhen Hospital of Peking University |
| 21 | VP07081 | OUT:K68 | 2007 | clinical | Xixiang People's Hospital |
| 22 | **VP07108** | O1:K36 | 2007 | clinical | Xixiang People's Hospital |
| 23 | **VP07111** | O2:K3 | 2007 | clinical | Xixiang People's Hospital |
| 24 | VP07112 | O1:K36 | 2007 | clinical | Shenzhen Center for Disease Control and Prevention |
| 25 | VP07118 | O1:K3 | 2007 | food | Shenzhen Center for Disease Control and Prevention |
| 26 | **VP07140** | O4:K8 | 2007 | clinical | Xixiang People's Hospital |
| 27 | **VP07141** | O1:K25 | 2007 | clinical | Xixiang People's Hospital |
| 28 | **VP07154** | O1:K25 | 2007 | clinical | Xixiang People's Hospital |
| 29 | **VP07181** | O1:K36 | 2007 | clinical | Xixiang People's Hospital |
| 30 | **VP07183** | O3:K29 | 2007 | clinical | Xixiang People's Hospital |
| 31 | **VP07184** | O4:K68 | 2007 | clinical | Xixiang People's Hospital |
| 32 | **VP07201** | O1:K25 | 2007 | clinical | Shenzhen Hospital of Peking University |
| 33 | **VP07202** | O4:K68 | 2007 | clinical | Xixiang People's Hospital |
| 34 | **VP07216** | O4:K8 | 2007 | clinical | Futian District Center for Disease Control and Prevention |
| 35 | VP07246 | OUT:K68 | 2007 | clinical | Xixiang People's Hospital |
| 36 | **VP07262** | O3:K6 | 2007 | clinical | Shenzhen Center for Disease Control and Prevention |
| 37 | VP08005 | OUT:K30 | 2008 | food | Shenzhen Center for Disease Control and Prevention |
| 38 | **VP08015** | O3:K6 | 2008 | clinical | Shenzhen Center for Disease Control and Prevention |
| 39 | VP08016 | O2:K3 | 2008 | clinical | Shenzhen Hospital of Peking University |
| 40 | **VP08018** | O4:K8 | 2008 | clinical | Shenzhen Hospital of Peking University |
| 41 | VP08040 | OUT:K13 | 2008 | clinical | Shenzhen Center for Disease Control and Prevention |
| 42 | **VP08041** | O4:K13 | 2008 | clinical | Yantian District Center for Disease Control and Prevention |
| 43 | **VP08071** | O1:K56 | 2008 | clinical | Longgang District Center for Disease Control and Prevention |
| 44 | VP08086 | O10:K32 | 2008 | food | Shenzhen Center for Disease Control and Prevention |
| 45 | VP08095 | O3:K32 | 2008 | food | Shenzhen Center for Disease Control and Prevention |
| 46 | VP08106 | O5:K32 | 2008 | food | Shenzhen Center for Disease Control and Prevention |
| 47 | VP08107 | O3:K48 | 2008 | clinical | Shenzhen Center for Disease Control and Prevention |
| 48 | **VP08109** | O8:K70 | 2008 | food | Shenzhen Center for Disease Control and Prevention |
| 49 | **VP08110** | O2:K3 | 2008 | food | Shenzhen Center for Disease Control and Prevention |
| 50 | **VP08111** | O5:K17 | 2008 | clinical | Longgang Central Hospital |
| 51 | **VP08115** | O1:K36 | 2008 | clinical | Shenzhen Hospital of Peking University |
| 52 | **VP08116** | O1:K56 | 2008 | clinical | Shenzhen Hospital of Peking University |
| 53 | VP08117 | O4:K8 | 2008 | clinical | Shenzhen Hospital of Peking University |
| 54 | VP08118 | O1:K8 | 2008 | clinical | Shenzhen Hospital of Peking University |
| 55 | VP08119 | O3:K6 | 2008 | clinical | Shenzhen Hospital of Peking University |
| 56 | VP08120 | O1:K56 | 2008 | food | Shenzhen Center for Disease Control and Prevention |
| 57 | VP08122 | O1:K41 | 2008 | clinical | Xixiang People's Hospital |
| 58 | **VP08125** | OUT:K6 | 2008 | clinical | Futian District Center for Disease Control and Prevention |
| 59 | **VP08139** | O2:K3 | 2008 | food | Shenzhen Center for Disease Control and Prevention |
| 60 | VP08144 | OUT:K6 | 2008 | food | Shenzhen Center for Disease Control and Prevention |
| 61 | VP08163 | O6:K18 | 2008 | food | Shenzhen Center for Disease Control and Prevention |
| 62 | **VP08181** | O3:K29 | 2008 | clinical | Xixiang People's Hospital |
| 63 | VP08182 | O4:K9 | 2008 | clinical | Xixiang People's Hospital |
| 64 | VP08183 | O4:K9 | 2008 | clinical | Xixiang People's Hospital |
| 65 | VP08189 | O1:K56 | 2008 | clinical | Xixiang People's Hospital |
| 66 | **VP08200** | O2:K3 | 2008 | clinical | Xixiang People's Hospital |
| 67 | VP08206 | O3:K6 | 2008 | clinical | Shenzhen Hospital of Peking University |
| 68 | VP08207 | O3:K6 | 2008 | clinical | Shenzhen Hospital of Peking University |
| 69 | **VP08213** | O4:K13 | 2008 | clinical | Futian District Center for Disease Control and Prevention |
| 70 | **VP08214** | O4:K13 | 2008 | clinical | Futian District Center for Disease Control and Prevention |
| 71 | **VP08215** | O4:K13 | 2008 | clinical | Futian District Center for Disease Control and Prevention |
| 72 | **VP08216** | O4:K13 | 2008 | clinical | Futian District Center for Disease Control and Prevention |
| 73 | **VP08220** | O4:K9 | 2008 | clinical | Xixiang People's Hospital |
| 74 | VP08243 | O1:K25 | 2008 | clinical | Xixiang People's Hospital |
| 75 | **VP08262** | O3:K6 | 2008 | food | Shenzhen Center for Disease Control and Prevention |
| 76 | **VP08277** | O1:K25 | 2008 | clinical | Xixiang People's Hospital |
| 77 | **VP08279** | O1:K56 | 2008 | food | Longgang District Center for Disease Control and Prevention |
| 78 | **VP08280** | O4:K8 | 2008 | food | Longgang District Center for Disease Control and Prevention |
| 79 | VP08283 | O1:K56 | 2008 | food | Longgang District Center for Disease Control and Prevention |
| 80 | VP08289 | O4:K42 | 2008 | food | Longgang District Center for Disease Control and Prevention |
| 81 | VP08298 | O4:K34 | 2008 | food | Longgang District Center for Disease Control and Prevention |
| 82 | **VP08300** | O1:K56 | 2008 | food | Longgang District Center for Disease Control and Prevention |
| 83 | VP08305 | O1:K56 | 2008 | food | Longgang District Center for Disease Control and Prevention |
| 84 | **VP08311** | O1:K56 | 2008 | clinical | Xixiang People's Hospital |
| 85 | **VP08315** | O1:K25 | 2008 | clinical | Xixiang People's Hospital |
| 86 | **VP08317** | O4:K13 | 2008 | clinical | Xixiang People's Hospital |
| 87 | VP08324 | O4:K9 | 2008 | clinical | Xixiang People's Hospital |
| 88 | VP08360 | O1:K56 | 2008 | clinical | Nanshan District Center for Disease Control and Prevention |
| 89 | VP08361 | O1:K56 | 2008 | clinical | Shenzhen Center for Disease Control and Prevention |
| 90 | **VP08371** | O3:K6 | 2008 | food | Shenzhen Center for Disease Control and Prevention |
| 91 | VP08374 | O3：K6 | 2008 | clinical | Xixiang People's Hospital |
| 92 | **VP08377** | O1:K56 | 2008 | clinical | Xixiang People's Hospital |
| 93 | VP08379 | O1:K25 | 2008 | clinical | Xixiang People's Hospital |
| 94 | VP08383 | O2:K3 | 2008 | clinical | Shenzhen Hospital of Peking University |
| 95 | **VP08398** | O3:K29 | 2008 | clinical | Luohu District Center for Disease Control and Prevention |
| 96 | VP09005 | O1:K17 | 2009 | food | Shenzhen Center for Disease Control and Prevention |
| 97 | VP09007 | O2:K20 | 2009 | food | Shenzhen Center for Disease Control and Prevention |
| 98 | VP09009 | O1:K25 | 2009 | food | Shenzhen Center for Disease Control and Prevention |
| 99 | VP09010 | O1:K25 | 2009 | food | Shenzhen Center for Disease Control and Prevention |
| 100 | VP09014 | O1:K6 | 2009 | clinical | Shenzhen Hospital of Peking University |
| 101 | **VP09015** | O3:K29 | 2009 | clinical | Longgang Central Hospital |
| 102 | **VP09018** | O3:K29 | 2009 | clinical | Xixiang People's Hospital |
| 103 | **VP09020** | O3:K6 | 2009 | clinical | Shenzhen Center for Disease Control and Prevention |
| 104 | **VP09021** | O1:K36 | 2009 | clinical | Longgang Central Hospital |
| 105 | VP09022 | O10:K6 | 2009 | clinical | Nanshan People's Hospital |
| 106 | **VP09030** | O4:K8 | 2009 | clinical | Longgang Central Hospital |
| 107 | VP09035 | OUT:K37 | 2009 | food | Shenzhen Center for Disease Control and Prevention |
| 108 | **VP09036** | O4:K8 | 2009 | clinical | Shenzhen Hospital of Peking University |
| 109 | VP09048 | O3:K29 | 2009 | clinical | Shenzhen Hospital of Peking University |
| 110 | VP09065 | O3:K8 | 2009 | food | Longgang District Center for Disease Control and Prevention |
| 111 | VP09076 | OUT:K6 | 2009 | clinical | Nanshan District Center for Disease Control and Prevention |
| 112 | VP09110 | O2:K6 | 2009 | food | Nanshan District Center for Disease Control and Prevention |
| 113 | VP09113 | O11:K36 | 2009 | clinical | Shenzhen Hospital of Peking University |
| 114 | **VP09118** | O2:K3 | 2009 | clinical | Xixiang People's Hospital |
| 115 | VP09144 | O3:K29 | 2009 | clinical | Xixiang People's Hospital |
| 116 | VP09145 | O3:K29 | 2009 | clinical | Xixiang People's Hospital |
| 117 | VP09151 | O3:K29 | 2009 | clinical | Xixiang People's Hospital |
| 118 | VP09159 | O5:K6 | 2009 | clinical | Nanshan District Center for Disease Control and Prevention |
| 119 | VP09175 | O1:K6 | 2009 | clinical | Nanshan District Center for Disease Control and Prevention |
| 120 | **VP09179** | O1:K56 | 2009 | clinical | Xixiang People's Hospital |
| 121 | VP09201 | O3:K29 | 2009 | clinical | Xixiang People's Hospital |
| 122 | **VP09202** | O1:K36 | 2009 | clinical | Xixiang People's Hospital |
| 123 | VP09216 | O3:K29 | 2009 | clinical | Xixiang People's Hospital |
| 124 | VP09227 | O11:K36 | 2009 | clinical | Nanshan District Center for Disease Control and Prevention |
| 125 | **VP09232** | O4:K8 | 2009 | clinical | Nanshan District Center for Disease Control and Prevention |
| 126 | VP09243 | O5:K68 | 2009 | clinical | Futian District Center for Disease Control and Prevention |
| 127 | VP09244 | O3:K6 | 2009 | clinical | Futian District Center for Disease Control and Prevention |
| 128 | **VP09254** | O4:K68 | 2009 | clinical | Baoan District Center for Disease Control and Prevention |
| 129 | VP09255 | O3:K29 | 2009 | clinical | Nanshan District Center for Disease Control and Prevention |
| 130 | VP09257 | O3:K29 | 2009 | clinical | Baoan District Center for Disease Control and Prevention |
| 131 | VP09259 | O3:K29 | 2009 | clinical | Baoan District Center for Disease Control and Prevention |
| 132 | VP09282 | O10:K19 | 2009 | food | Shenzhen Center for Disease Control and Prevention |
| 133 | VP09286 | O2:K17 | 2009 | food | Shenzhen Center for Disease Control and Prevention |
| 134 | **VP09302** | O2:K3 | 2009 | clinical | Shenzhen Hospital of Peking University |
| 135 | VP09317 | O4:K9 | 2009 | clinical | Songgang People's Hospital |
| 136 | VP09319 | O4:K9 | 2009 | food | Songgang People's Hospital |
| 137 | VP09322 | O5:K68 | 2009 | clinical | Shenzhen Center for Disease Control and Prevention |
| 138 | VP09329 | O11:K36 | 2009 | clinical | Xixiang People's Hospital |
| 139 | VP09333 | O5:K68 | 2009 | clinical | Shenzhen Center for Disease Control and Prevention |
| 140 | VP09334 | O5:K68 | 2009 | clinical | Shenzhen Center for Disease Control and Prevention |
| 141 | VP09360 | O5:K68 | 2009 | clinical | Shenzhen Center for Disease Control and Prevention |
| 142 | VP09361 | O5:K68 | 2009 | clinical | Shenzhen Center for Disease Control and Prevention |
| 143 | VP09363 | O3:K29 | 2009 | clinical | Xixiang People's Hospital |
| 144 | VP09366 | O3:K29 | 2009 | clinical | Xixiang People's Hospital |
| 145 | VP09367 | O3:K29 | 2009 | clinical | Xixiang People's Hospital |
| 146 | VP09374 | O5:K68 | 2009 | clinical | Shenzhen Center for Disease Control and Prevention |
| 147 | VP09375 | O5:K68 | 2009 | clinical | Shenzhen Center for Disease Control and Prevention |
| 148 | VP09422 | O4:K13 | 2009 | clinical | Shenzhen Center for Disease Control and Prevention |
| 149 | VP09425 | O3:K29 | 2009 | clinical | Xixiang People's Hospital |
| 150 | VP09427 | O3:K29 | 2009 | clinical | Xixiang People's Hospital |
| 151 | VP09428 | O3:K29 | 2009 | clinical | Xixiang People's Hospital |
| 152 | VP09430 | O3:K29 | 2009 | clinical | Xixiang People's Hospital |
| 153 | VP09435 | O3:K29 | 2009 | clinical | Xixiang People's Hospital |
| 154 | VP09440 | O3:K29 | 2009 | clinical | Xixiang People's Hospital |
| 155 | VP09453 | O10:K25 | 2009 | food | Nanshan District Center for Disease Control and Prevention |
| 156 | VP09455 | O1:K42 | 2009 | food | Nanshan District Center for Disease Control and Prevention |
| 157 | VP09456 | O3:K29 | 2009 | clinical | Longgang Central Hospital |
| 158 | VP09460 | O3:K29 | 2009 | clinical | Xixiang People's Hospital |
| 159 | **VP09467** | O3:K6 | 2009 | food | Foshan Center for Disease Control and Prevention |
| 160 | **VP09469** | O3:K29 | 2009 | clinical | Longhua People's Hospital |
| 161 | VP09471 | O2:K3 | 2009 | clinical | Shenzhen Center for Disease Control and Prevention |
| 162 | VP10008 | O2:K28 | 2010 | clinical | Shenzhen Center for Disease Control and Prevention |
| 163 | **VP10010** | O1:K25 | 2010 | food | Nanshan District Center for Disease Control and Prevention |
| 164 | VP10012 | O2:K28 | 2010 | clinical | Shenzhen Center for Disease Control and Prevention |
| 165 | VP10016 | O2:K3 | 2010 | clinical | Foshan Center for Disease Control and Prevention |
| 166 | VP10023 | O5:K24 | 2010 | food | Foshan Center for Disease Control and Prevention |
| 167 | VP10033 | O2:K3 | 2010 | clinical | Xixiang People's Hospital |
| 168 | VP10036 | O2:K3 | 2010 | clinical | Xixiang People's Hospital |
| 169 | **VP10067** | O4:K8 | 2010 | clinical | Xixiang People's Hospital |
| 170 | VP10081 | O11:K36 | 2010 | clinical | Shenzhen Center for Disease Control and Prevention |
| 171 | VP10090 | O3:K29 | 2010 | clinical | Xixiang People's Hospital |
| 172 | VP10093 | O3:K29 | 2010 | clinical | Xixiang People's Hospital |
| 173 | VP10094 | OUT:K6 | 2010 | clinical | Xixiang People's Hospital |
| 174 | VP10105 | K29 | 2010 | clinical | Longgang Central Hospital |
| 175 | **VP10106** | O3:K6 | 2010 | clinical | Longgang Central Hospital |
| 176 | **VP10107** | O1:K36 | 2010 | clinical | Longgang Central Hospital |
| 177 | VP10113 | O1:K56 | 2010 | clinical | Xixiang People's Hospital |
| 178 | VP10114 | O1:K68 | 2010 | clinical | Xixiang People's Hospital |
| 179 | **VP10118** | O1:K56 | 2010 | clinical | Xixiang People's Hospital |
| 180 | **VP10125** | O3:K6 | 2010 | clinical | Baoan District Center for Disease Control and Prevention |
| 181 | **VP10133** | O1:K56 | 2010 | clinical | Luohu District Center for Disease Control and Prevention |
| 182 | **VP10134** | O1:K36 | 2010 | clinical | Luohu District Center for Disease Control and Prevention |
| 183 | VP10135 | O1:K56 | 2010 | clinical | Longgang Central Hospital |
| 184 | **VP10137** | O1:K36 | 2010 | clinical | Luohu District Center for Disease Control and Prevention |
| 185 | **VP10139** | O1:K36 | 2010 | clinical | Luohu District Center for Disease Control and Prevention |
| 186 | **VP10140** | O1:K36 | 2010 | clinical | Luohu District Center for Disease Control and Prevention |
| 187 | VP10142 | O1:K56 | 2010 | clinical | Luohu District Center for Disease Control and Prevention |
| 188 | **VP10143** | O1:K36 | 2010 | clinical | Luohu District Center for Disease Control and Prevention |
| 189 | **VP10146** | O1:K36 | 2010 | clinical | Luohu District Center for Disease Control and Prevention |
| 190 | VP10150 | O10:K6 | 2010 | clinical | Luohu District Center for Disease Control and Prevention |
| 191 | VP10151 | O1:K56 | 2010 | clinical | Luohu District Center for Disease Control and Prevention |
| 192 | VP10152 | O1:K56 | 2010 | clinical | Luohu District Center for Disease Control and Prevention |
| 193 | **VP10153** | O1:K56 | 2010 | clinical | Luohu District Center for Disease Control and Prevention |
| 194 | VP10154 | O1:K56 | 2010 | clinical | Luohu District Center for Disease Control and Prevention |
| 195 | VP10155 | O1:K56 | 2010 | clinical | Luohu District Center for Disease Control and Prevention |
| 196 | **VP10159** | O1:K56 | 2010 | clinical | Longgang Central Hospital |
| 197 | VP10160 | O1:K56 | 2010 | clinical | Luohu District Center for Disease Control and Prevention |
| 198 | VP10162 | O1:K56 | 2010 | clinical | Longgang Central Hospital |
| 199 | VP10165 | O1:K56 | 2010 | clinical | Longgang Central Hospital |
| 200 | VP10166 | O1:K56 | 2010 | clinical | Xixiang People's Hospital |
| 201 | VP10167 | O1:K56 | 2010 | clinical | Xixiang People's Hospital |
| 202 | VP10173 | O1:K56 | 2010 | clinical | Xixiang People's Hospital |
| 203 | VP10175 | O1:K56 | 2010 | clinical | Xixiang People's Hospital |
| 204 | **VP10180** | O4:K8 | 2010 | clinical | Futian District Center for Disease Control and Prevention |
| 205 | **VP10183** | O1:K36 | 2010 | clinical | Longgang People's Hospital |
| 206 | VP10185 | O1:K56 | 2010 | clinical | Xixiang People's Hospital |
| 207 | VP10186 | O1:K56 | 2010 | clinical | Xixiang People's Hospital |
| 208 | VP10187 | O1:K56 | 2010 | clinical | Xixiang People's Hospital |
| 209 | VP10188 | O1:K56 | 2010 | clinical | Xixiang People's Hospital |
| 210 | VP10193 | O5:K68 | 2010 | clinical | Xixiang People's Hospital |
| 211 | VP10216 | O1:K56 | 2010 | clinical | Xixiang People's Hospital |
| 212 | **VP10217** | O1:K56 | 2010 | clinical | Guangming People's Hospital |
| 213 | **VP10218** | O2:K3 | 2010 | food | Shenzhen Center for Disease Control and Prevention |
| 214 | VP10224 | O2:K34 | 2010 | food | Shenzhen Center for Disease Control and Prevention |
| 215 | VP10225 | O1:K36 | 2010 | clinical | Xixiang People's Hospital |
| 216 | **VP10226** | O1:K36 | 2010 | clinical | Longgang Central Hospital |
| 217 | VP10227 | O1:K56 | 2010 | clinical | Xixiang People's Hospital |
| 218 | VP10230 | OUT:K6 | 2010 | food | Shenzhen Center for Disease Control and Prevention |
| 219 | VP10231 | O1:K56 | 2010 | clinical | Longgang Central Hospital |
| 220 | **VP10233** | O1:K36 | 2010 | clinical | Longgang Central Hospital |
| 221 | **VP10236** | O1:K36 | 2010 | clinical | Guangming People's Hospital |
| 222 | **VP10237** | O4:K68 | 2010 | clinical | Guangming People's Hospital |
| 223 | **VP10238** | O1:K36 | 2010 | clinical | Guangming People's Hospital |
| 224 | **VP10241** | O4:K68 | 2010 | clinical | Longgang People's Hospital |
| 225 | VP10245 | O3:K29 | 2010 | clinical | Xixiang People's Hospital |
| 226 | **VP10250** | O3:K29 | 2010 | clinical | Xixiang People's Hospital |
| 227 | **VP10251** | O4:K68 | 2010 | clinical | Xixiang People's Hospital |
| 228 | **VP10253** | O3:K6 | 2010 | clinical | Shenzhen Center for Disease Control and Prevention |
| 229 | VP10254 | O3:K6 | 2010 | clinical | Guangming People's Hospital |
| 230 | VP10256 | O1:K56 | 2010 | clinical | Longgang Central Hospital |
| 231 | VP10257 | O3:K29 | 2010 | clinical | Longgang Central Hospital |
| 232 | **VP10260** | O4:K68 | 2010 | clinical | Guangming People's Hospital |
| 233 | **VP10263** | O4:K8 | 2010 | clinical | Guangming People's Hospital |
| 234 | VP10281 | O1:K56 | 2010 | clinical | Xixiang People's Hospital |
| 235 | VP10285 | O1:K56 | 2010 | clinical | Xixiang People's Hospital |
| 236 | **VP10288** | O1:K56 | 2010 | clinical | Futian District Center for Disease Control and Prevention |
| 237 | **VP10304** | O1:K36 | 2010 | clinical | Longgang Central Hospital |
| 238 | **VP10316** | O4:K68 | 2010 | clinical | Longgang Central Hospital |
| 239 | VP10323 | O1:K56 | 2010 | clinical | Xixiang People's Hospital |
| 240 | VP10324 | O1:K56 | 2010 | clinical | Xixiang People's Hospital |
| 241 | **VP10334** | O1:K56 | 2010 | clinical | Shenzhen Hospital of Peking University |
| 242 | VP10336 | O3:K29 | 2010 | clinical | Shenzhen Hospital of Peking University |
| 243 | **VP10362** | O2:K3 | 2010 | clinical | Xixiang People's Hospital |
| 244 | **VP10364** | O4:K68 | 2010 | clinical | Xixiang People's Hospital |
| 245 | VP10370 | O3:K29 | 2010 | clinical | Baoan District Center for Disease Control and Prevention |
| 246 | VP10371 | O3:K29 | 2010 | clinical | Baoan District Center for Disease Control and Prevention |
| 247 | VP10372 | O3:K29 | 2010 | clinical | Baoan District Center for Disease Control and Prevention |
| 248 | VP10373 | O3:K29 | 2010 | clinical | Baoan District Center for Disease Control and Prevention |
| 249 | VP10418 | O3:K29 | 2010 | clinical | Longgang Central Hospital |
| 250 | VP10441 | O1:K56 | 2010 | clinical | Guangming People's Hospital |
| 251 | **VP10444** | O2:K3 | 2010 | clinical | Longgang Central Hospital |
| 252 | **VP10446** | O1:K25 | 2010 | food | Longgang Central Hospital |
| 253 | SPVP110023 | O2:K17 | 2011 | food | Baoan District Center for Disease Control and Prevention |
| 254 | SPVP110026 | O6:K18 | 2011 | food | Guangming District Center for Disease Control and Prevention |
| 255 | SPVP110028 | O6:K18 | 2011 | food | Futian District Center for Disease Control and Prevention |
| 256 | SPVP110047 | O6:K18 | 2011 | food | Guangming District Center for Disease Control and Prevention |
| 257 | SPVP110054 | O6:K18 | 2011 | food | Guangming District Center for Disease Control and Prevention |
| 258 | SPVP110064 | O1:K18 | 2011 | food | Nanshan District Center for Disease Control and Prevention |
| 259 | SPVP110070 | O6:K18 | 2011 | food | Longgang District Center for Disease Control and Prevention |
| 260 | SPVP110072 | O6:K18 | 2011 | food | Longgang District Center for Disease Control and Prevention |
| 261 | SPVP110097 | O3:K5 | 2011 | food | Futian District Center for Disease Control and Prevention |
| 262 | SPVP110102 | O6:K18 | 2011 | food | Futian District Center for Disease Control and Prevention |
| 263 | **VP11052** | O2:K3 | 2011 | clinical | Xixiang People's Hospital |
| 264 | **VP11073** | O4:K9 | 2011 | clinical | Xixiang People's Hospital |
| 265 | **VP11121** | O4:K68 | 2011 | clinical | Xixiang People's Hospital |
| 266 | **VP11144** | O3:K6 | 2011 | clinical | Nanshan District Center for Disease Control and Prevention |
| 267 | **VP11149** | O4:K8 | 2011 | clinical | Xixiang People's Hospital |
| 268 | **VP11176** | O4:K68 | 2011 | clinical | Xixiang People's Hospital |
| 269 | **VP11180** | O1:K25 | 2011 | clinical | Xixiang People's Hospital |
| 270 | **VP11196** | O4:K68 | 2011 | clinical | Xixiang People's Hospital |
| 271 | VP11221 | O4:K6 | 2011 | clinical | Yantian District Center for Disease Control and Prevention |
| 272 | **VP11254** | O2:K3 | 2011 | food | Nanshan District Center for Disease Control and Prevention |
| 273 | **VP11284** | O4:K8 | 2011 | clinical | Longgang Central Hospital |
| 274 | **VP11298** | O4:K8 | 2011 | clinical | Xixiang People's Hospital |
| 275 | **VP11313** | O3:K6 | 2011 | clinical | Xixiang People's Hospital |
| 276 | **VP12010** | O1:K56 | 2012 | clinical | Longgang People's Hospital |
| 277 | **VP12016** | O4:K68 | 2012 | clinical | Nanshan District Center for Disease Control and Prevention |
| 278 | VP12017 | O1:K36 | 2012 | clinical | Nanshan District Center for Disease Control and Prevention |
| 279 | VP12030 | O1:K6 | 2012 | clinical | Nanshan District Center for Disease Control and Prevention |
| 280 | **VP12031** | O4:K8 | 2012 | clinical | Futian District Center for Disease Control and Prevention |
| 281 | VP12034 | O2:K3 | 2012 | clinical | Xixiang People's Hospital |
| 282 | **VP12051** | O3:K6 | 2012 | clinical | Xixiang People's Hospital |
| 283 | VP12061 | O2:K3 | 2012 | clinical | Longhua People's Hospital |
| 284 | **VP12078** | O3:K6 | 2012 | clinical | Longhua People's Hospital |
| 285 | VP12082 | OUT:K29 | 2012 | clinical | Longgang District Center for Disease Control and Prevention |
| 286 | **VP12083** | O3:K29 | 2012 | clinical | Baoan Central Hospital |
| 287 | **VP12087** | O4:K8 | 2012 | clinical | Futian District Center for Disease Control and Prevention |
| 288 | **VP12103** | O4:K8 | 2012 | clinical | Xixiang People's Hospital |
| 289 | **VP12111** | O3:K6 | 2012 | clinical | Xixiang People's Hospital |
| 290 | **VP12118** | O4:K8 | 2012 | clinical | Nanshan District Center for Disease Control and Prevention |
| 291 | **VP12119** | O4:K8 | 2012 | clinical | Shajng People's Hospital |
| 292 | **VP12130** | O4:K9 | 2012 | clinical | Shenzhen Center for Disease Control and Prevention |
| 293 | **VP12163** | O4:K9 | 2012 | clinical | Shenzhen Center for Disease Control and Prevention |
| 294 | **VP12171** | O4:K8 | 2012 | clinical | Shenzhen Center for Disease Control and Prevention |
| 295 | **VP12174** | O3:K29 | 2012 | clinical | Shenzhen Center for Disease Control and Prevention |
| 296 | VP12187 | O2:K3 | 2012 | clinical | Longgang Central Hospital |
| 297 | **VP12196** | O4:K9 | 2012 | clinical | Shenzhen Center for Disease Control and Prevention |
| 298 | **VP12201** | O1:K56 | 2012 | clinical | Shenzhen Center for Disease Control and Prevention |
| 299 | VP12230 | O1:K6 | 2012 | clinical | Longgang People's Hospital |
| 300 | VP12232 | O4:K9 | 2012 | clinical | Shenzhen Center for Disease Control and Prevention |
| 301 | **VP12233** | O4:K9 | 2012 | clinical | Shenzhen Center for Disease Control and Prevention |
| 302 | **VP12236** | O3:K6 | 2012 | clinical | Shenzhen Hospital of Peking University |
| 303 | VP12237 | O4:K9 | 2012 | clinical | Shenzhen Hospital of Peking University |
| 304 | **VP13011** | O2:K3 | 2013 | clinical | Shenzhen Center for Disease Control and Prevention |
| 305 | **VP13013** | O4:K8 | 2013 | clinical | Shenzhen Center for Disease Control and Prevention |
| 306 | **VP13018** | O4:K8 | 2013 | clinical | Shenzhen Center for Disease Control and Prevention |
| 307 | **VP13030** | O3:K6 | 2013 | clinical | Shenzhen Center for Disease Control and Prevention |
| 308 | **VP13058** | O1:K56 | 2013 | clinical | Shajng People's Hospital |
| 309 | **VP13083** | O1:K56 | 2013 | clinical | Nanshan People's Hospital |
| 310 | VP13092 | O1:K9 | 2013 | clinical | Longhua People's Hospital |
| 311 | **VP13104** | O4:K12 | 2013 | clinical | Songgang People's Hospital |
| 312 | VP13121 | O1:K25 | 2013 | food | Shenzhen Center for Disease Control and Prevention |
| 313 | VP14096 | O4:K6 | 2014 | clinical | Longgang People's Hospital |
| 314 | VP15002 | O10:K67 | 2015 | clinical | Shenzhen Hospital of Peking University |
| 315 | VP15003 | O4:K6 | 2015 | clinical | Shenzhen Hospital of Peking University |
| 316 | **VP15004** | O3:K6 | 2015 | clinical | Shenzhen Center for Disease Control and Prevention |
| 317 | **VP15023** | O4:K8 | 2015 | clinical | Longhua People's Hospital |
| 318 | **VP15112** | OUT:K6 | 2015 | clinical | Xixiang People's Hospital |
| 319 | **VP15113** | O1:K56 | 2015 | clinical | Songgang People's Hospital |
| 320 | **VP15115** | O1:K56 | 2015 | clinical | Songgang People's Hospital |
| 321 | **vp15126** | O3:K6 | 2015 | food | Shenzhen Center for Disease Control and Prevention |
| 322 | **VP15141** | O4:K8 | 2015 | clinical | Xixiang People's Hospital |
| 323 | **VP15159** | O4:K8 | 2015 | clinical | Xixiang People's Hospital |
| 324 | **VP15161** | O3:K6 | 2015 | clinical | Xixiang People's Hospital |
| 325 | **VP15166** | O4:K8 | 2015 | clinical | Nanshan People's Hospital |
| 326 | VP15170 | O4:K6 | 2015 | clinical | Nanshan People's Hospital |
| 327 | VP15178 | O3:K6 | 2015 | clinical | Xixiang People's Hospital |
| 328 | VP15204 | O1:K6 | 2015 | clinical | Shenzhen Center for Disease Control and Prevention |
| 329 | **VP15205** | O4:K68 | 2015 | clinical | Xixiang People's Hospital |
| 330 | **VP16107** | O1:K36 | 2016 | clinical | Baoan Central Hospital |
| 331 | **VP16114** | O4:K8 | 2016 | clinical | Songgang People's Hospital |
| 332 | **VP16135** | O1:K56 | 2016 | clinical | Baoan Central Hospital |
| 333 | VP16144 | O4:K6 | 2016 | clinical | Nanshan District Center for Disease Control and Prevention |
| 334 | **VP16163** | O3:K6 | 2016 | clinical | Shenzhen Center for Disease Control and Prevention |
| 335 | **VP16185** | O1:K56 | 2016 | clinical | Guangming People's Hospital |
| 336 | **VP16194** | O3:K6 | 2016 | clinical | Longgang People's Hospital |
| 337 | **VP16200** | O1:K36 | 2016 | clinical | Shenzhen Center for Disease Control and Prevention |
| 338 | **VP17002** | O3:K6 | 2017 | food | Shenzhen Center for Disease Control and Prevention |
| 339 | **VP17013** | O4:K8 | 2017 | clinical | Longhua District Center for Disease Control and Prevention |
| 340 | **VP17040** | O3:K6 | 2017 | clinical | Guangming District Center for Disease Control and Prevention |
| 341 | **VP17041** | O3:K6 | 2017 | clinical | Longgang District Center for Disease Control and Prevention |
| 342 | **VP17043** | O4:K8 | 2017 | clinical | Yantian District Center for Disease Control and Prevention |
| 343 | VP17052 | O4:K6 | 2017 | clinical | Shajng People's Hospital |
| 344 | VP17062 | O4:K6 | 2017 | clinical | Dapeng District Center for Disease Control and Prevention |
| 345 | VP17069 | O1:K8 | 2017 | clinical | Dapeng District Center for Disease Control and Prevention |
| 346 | VP17076 | O1:K6 | 2017 | clinical | Dapeng District Center for Disease Control and Prevention |
| 347 | VP17081 | O4:K6 | 2017 | clinical | Dapeng District Center for Disease Control and Prevention |
| 348 | **VP17091** | O1:K36 | 2017 | food | Longgang District Center for Disease Control and Prevention |
| 349 | **VP17092** | O1:K25 | 2017 | clinical | Nanshan District Center for Disease Control and Prevention |
| 350 | **VP17104** | O4:K8 | 2017 | clinical | Baoan Central Hospital |
| 351 | **VP17108** | O3:K6 | 2017 | clinical | Songgang People's Hospital |
| 352 | **VP17117** | O3:K6 | 2017 | clinical | Songgang People's Hospital |
| 353 | **VP17126** | O3:K6 | 2017 | clinical | Baoan Central Hospital |
| 354 | **VP17135** | O3:K6 | 2017 | clinical | Baoan Central Hospital |
| 355 | **VP17144** | O3:K6 | 2017 | clinical | Baoan Central Hospital |
| 356 | VP17151 | O1:K54 | 2017 | clinical | Shenzhen Hospital of Peking University |
| 357 | VP18012 | O4:K6 | 2018 | clinical | Baoan Central Hospital |
| 358 | VP18019 | O3:K6 | 2018 | clinical | Songgang People's Hospital |
| 359 | VP18088 | OUT:K8 | 2018 | food | Nanshan District Center for Disease Control and Prevention |

* Bold typeface indicates 165 strains among 359 strains that overlap with the 418 *V. parahaemolyticus* strains.

| **TABLE S3** **\|** The general information for the 236 *V. parahaemolyticus* strains. | | | | | |
| --- | --- | --- | --- | --- | --- |
| **No.** | **Sample ID** | **Serotype** | **Year** | **Source** | **Location** |
| 1 | VP04079 | O3:KUT | 2004 | clinical | Luohu District Center for Disease Control and Prevention |
| 2 | VP04080 | O3:KUT | 2004 | clinical | Luohu District Center for Disease Control and Prevention |
| 3 | VP04114 | O1:KUT | 2004 | clinical | Nanshan District Center for Disease Control and Prevention |
| 4 | VP04128 | O4:KUT | 2004 | clinical | Nanshan District Center for Disease Control and Prevention |
| 5 | VP04141 | OUT:KUT | 2004 | clinical | Nanshan District Center for Disease Control and Prevention |
| 6 | VP05237 | OUT:KUT | 2005 | clinical | Luohu District Center for Disease Control and Prevention |
| 7 | VP05239 | OUT:KUT | 2005 | clinical | Luohu District Center for Disease Control and Prevention |
| 8 | VP05258 | OUT:KUT | 2005 | clinical | Luohu District Center for Disease Control and Prevention |
| 9 | VP05260 | O1:KUT | 2005 | clinical | Luohu District Center for Disease Control and Prevention |
| 10 | VP05265 | O1:KUT | 2005 | clinical | Shenzhen Center for Disease Control and Prevention |
| 11 | VP06019 | O1:KUT | 2006 | clinical | Nanshan District Center for Disease Control and Prevention |
| 12 | VP06020 | O1:KUT | 2006 | clinical | Nanshan District Center for Disease Control and Prevention |
| 13 | VP06026 | O2:KUT | 2006 | food | Luohu District Center for Disease Control and Prevention |
| 14 | VP06027 | O3:KUT | 2006 | clinical | Luohu District Center for Disease Control and Prevention |
| 15 | VP06060 | O1:KUT | 2006 | clinical | Yantian District Center for Disease Control and Prevention |
| 16 | VP06070 | OUT:KUT | 2006 | clinical | Luohu District Center for Disease Control and Prevention |
| 17 | VP06071 | O1:KUT | 2006 | food | Luohu District Center for Disease Control and Prevention |
| 18 | VP06098 | O1:KUT | 2006 | clinical | Luohu District Center for Disease Control and Prevention |
| 19 | VP06099 | O1:KUT | 2006 | clinical | Luohu District Center for Disease Control and Prevention |
| 20 | VP06100 | OUT:KUT | 2006 | clinical | Luohu District Center for Disease Control and Prevention |
| 21 | VP06101 | O1:KUT | 2006 | clinical | Luohu District Center for Disease Control and Prevention |
| 22 | VP06102 | O9:KUT | 2006 | clinical | Luohu District Center for Disease Control and Prevention |
| 23 | VP06103 | OUT:KUT | 2006 | clinical | Luohu District Center for Disease Control and Prevention |
| 24 | VP06104 | O6:KUT | 2006 | clinical | Luohu District Center for Disease Control and Prevention |
| 25 | VP06105 | OUT:KUT | 2006 | clinical | Luohu District Center for Disease Control and Prevention |
| 26 | VP06106 | O1:KUT | 2006 | clinical | Luohu District Center for Disease Control and Prevention |
| 27 | VP06107 | O1:KUT | 2006 | clinical | Luohu District Center for Disease Control and Prevention |
| 28 | VP06108 | O1:KUT | 2006 | clinical | Luohu District Center for Disease Control and Prevention |
| 29 | VP07031 | O1:KUT | 2007 | clinical | Nanshan District Center for Disease Control and Prevention |
| 30 | VP07050 | OUT:KUT | 2007 | clinical | Xixiang People's Hospital |
| 31 | VP07106 | O1:KUT | 2007 | clinical | Xixiang People's Hospital |
| 32 | VP07194 | OUT:KUT | 2007 | clinical | Xixiang People's Hospital |
| 33 | VP08039 | O10:KUT | 2008 | food | Longgang District Center for Disease Control and Prevention |
| 34 | VP08057 | O3:KUT | 2008 | clinical | Longgang Central Hospital |
| 35 | VP08085 | O2:KUT | 2008 | clinical | Shenzhen Center for Disease Control and Prevention |
| 36 | VP08091 | OUT:KUT | 2008 | food | Shenzhen Center for Disease Control and Prevention |
| 37 | VP08098 | O5:KUT | 2008 | clinical | Shenzhen Center for Disease Control and Prevention |
| 38 | VP08099 | O4:KUT | 2008 | food | Shenzhen Center for Disease Control and Prevention |
| 39 | VP08102 | O3:KUT | 2008 | food | Shenzhen Center for Disease Control and Prevention |
| 40 | VP08113 | O5:KUT | 2008 | food | Shenzhen Center for Disease Control and Prevention |
| 41 | VP08140 | O2:KUT | 2008 | food | Shenzhen Center for Disease Control and Prevention |
| 42 | VP08141 | O1:KUT | 2008 | clinical | Shenzhen Center for Disease Control and Prevention |
| 43 | VP08176 | O1:KUT | 2008 | clinical | Xixiang People's Hospital |
| 44 | VP08178 | O1:KUT | 2008 | clinical | Xixiang People's Hospital |
| 45 | VP08186 | O1:KUT | 2008 | clinical | Xixiang People's Hospital |
| 46 | VP08192 | O1:KUT | 2008 | clinical | Xixiang People's Hospital |
| 47 | VP08195 | O1:KUT | 2008 | clinical | Xixiang People's Hospital |
| 48 | VP08196 | O1:KUT | 2008 | clinical | Xixiang People's Hospital |
| 49 | VP08218 | O1:KUT | 2008 | clinical | Xixiang People's Hospital |
| 50 | VP08219 | O1:KUT | 2008 | clinical | Xixiang People's Hospital |
| 51 | VP08222 | O4:KUT | 2008 | clinical | Xixiang People's Hospital |
| 52 | VP08223 | O1:KUT | 2008 | clinical | Xixiang People's Hospital |
| 53 | VP08224 | O1:KUT | 2008 | clinical | Xixiang People's Hospital |
| 54 | VP08244 | O1:KUT | 2008 | clinical | Xixiang People's Hospital |
| 55 | VP08282 | O4:KUT | 2008 | food | Longgang District Center for Disease Control and Prevention |
| 56 | VP08285 | O1:KUT | 2008 | food | Longgang District Center for Disease Control and Prevention |
| 57 | VP08286 | O2:KUT | 2008 | clinical | Longgang District Center for Disease Control and Prevention |
| 58 | VP08292 | O2:KUT | 2008 | food | Longgang District Center for Disease Control and Prevention |
| 59 | VP08293 | O4:KUT | 2008 | food | Longgang District Center for Disease Control and Prevention |
| 60 | VP08294 | 05:KUT | 2008 | food | Longgang District Center for Disease Control and Prevention |
| 61 | VP08295 | O1:KUT | 2008 | food | Longgang District Center for Disease Control and Prevention |
| 62 | VP08301 | O1:KUT | 2008 | food | Longgang District Center for Disease Control and Prevention |
| 63 | VP08302 | O1:KUT | 2008 | food | Longgang District Center for Disease Control and Prevention |
| 64 | VP08304 | O1:KUT | 2008 | food | Longgang District Center for Disease Control and Prevention |
| 65 | VP08306 | O1:KUT | 2008 | food | Longgang District Center for Disease Control and Prevention |
| 66 | VP08309 | O8:KUT | 2008 | food | Longgang District Center for Disease Control and Prevention |
| 67 | VP08312 | O2:KUT | 2008 | food | Longgang District Center for Disease Control and Prevention |
| 68 | VP08313 | O1:KUT | 2008 | clinical | Xixiang People's Hospital |
| 69 | VP08321 | O1:KUT | 2008 | clinical | Xixiang People's Hospital |
| 70 | VP08325 | O1:KUT | 2008 | clinical | Xixiang People's Hospital |
| 71 | VP08327 | O1:KUT | 2008 | clinical | Xixiang People's Hospital |
| 72 | VP08349 | O2:KUT | 2008 | food | Longgang District Center for Disease Control and Prevention |
| 73 | VP08406 | O1:KUT | 2008 | food | Shenzhen Center for Disease Control and Prevention |
| 74 | VP08410 | O2:KUT | 2008 | food | Shenzhen Center for Disease Control and Prevention |
| 75 | VP08412 | O1:KUT | 2008 | food | Shenzhen Center for Disease Control and Prevention |
| 76 | VP08413 | O1:KUT | 2008 | food | Shenzhen Center for Disease Control and Prevention |
| 77 | VP09003 | O3:KUT | 2009 | food | Luohu District Center for Disease Control and Prevention |
| 78 | VP09006 | O1:KUT | 2009 | food | Shenzhen Center for Disease Control and Prevention |
| 79 | VP09011 | O3:KUT | 2009 | clinical | Shenzhen Hospital of Peking University |
| 80 | VP09034 | OUT:KUT | 2009 | food | Shenzhen Center for Disease Control and Prevention |
| 81 | VP09087 | O4:KUT | 2009 | clinical | Xixiang People's Hospital |
| 82 | VP09097 | O1:KUT | 2009 | food | Nanshan District Center for Disease Control and Prevention |
| 83 | VP09207 | O6:KUT | 2009 | clinical | Shenzhen Center for Disease Control and Prevention |
| 84 | VP09285 | O10:KUT | 2009 | food | Shenzhen Center for Disease Control and Prevention |
| 85 | VP09287 | O5:KUT | 2009 | food | Shenzhen Center for Disease Control and Prevention |
| 86 | VP09290 | O2:KUT | 2009 | clinical | Shenzhen Center for Disease Control and Prevention |
| 87 | VP09292 | O3:KUT | 2009 | clinical | Shenzhen Center for Disease Control and Prevention |
| 88 | VP09293 | O2:KUT | 2009 | food | Shenzhen Center for Disease Control and Prevention |
| 89 | VP09441 | O3:KUT | 2009 | clinical | Xixiang People's Hospital |
| 90 | VP09449 | O11:KUT | 2009 | clinical | Shenzhen Center for Disease Control and Prevention |
| 91 | VP09451 | O10:KUT | 2009 | food | Nanshan District Center for Disease Control and Prevention |
| 92 | VP09454 | O1:KUT | 2009 | food | Nanshan District Center for Disease Control and Prevention |
| 93 | VP09464 | O1:KUT | 2009 | clinical | Xixiang People's Hospital |
| 94 | VP09465 | O4:KUT | 2009 | food | Shenzhen Center for Disease Control and Prevention |
| 95 | VP09475 | O1:KUT | 2009 | clinical | Xixiang People's Hospital |
| 96 | VP09481 | O1:KUT | 2009 | clinical | Xixiang People's Hospital |
| 97 | VP10005 | O1:KUT | 2010 | food | Shenzhen Center for Disease Control and Prevention |
| 98 | VP10006 | O2:KUT | 2010 | food | Nanshan District Center for Disease Control and Prevention |
| 99 | VP10015 | O2:KUT | 2010 | clinical | Shenzhen Center for Disease Control and Prevention |
| 100 | VP10035 | O3:KUT | 2010 | clinical | Xixiang People's Hospital |
| 101 | VP10055 | O1:KUT | 2010 | clinical | Shenzhen Center for Disease Control and Prevention |
| 102 | VP10057 | O2:KUT | 2010 | clinical | Foshan Center for Disease Control and Prevention |
| 103 | VP10059 | O5:KUT | 2010 | clinical | Foshan Center for Disease Control and Prevention |
| 104 | VP10060 | O2:KUT | 2010 | clinical | Foshan Center for Disease Control and Prevention |
| 105 | VP10061 | OUT:KUT | 2010 | clinical | Foshan Center for Disease Control and Prevention |
| 106 | VP10062 | O4:KUT | 2010 | clinical | Foshan Center for Disease Control and Prevention |
| 107 | VP10072 | O4:KUT | 2010 | clinical | Nanshan District Center for Disease Control and Prevention |
| 108 | VP10082 | O3:KUT | 2010 | clinical | Xixiang People's Hospital |
| 109 | VP10087 | O2:KUT | 2010 | clinical | Xixiang People's Hospital |
| 110 | VP10168 | O1:KUT | 2010 | clinical | Xixiang People's Hospital |
| 111 | VP10189 | O1:KUT | 2010 | clinical | Xixiang People's Hospital |
| 112 | VP10220 | O2:KUT | 2010 | food | Shenzhen Center for Disease Control and Prevention |
| 113 | VP10222 | O2:KUT | 2010 | food | Shenzhen Center for Disease Control and Prevention |
| 114 | VP10490 | O1:KUT | 2010 | clinical | Longgang People's Hospital |
| 115 | SPVP110001 | O2:KUT | 2011 | food | Guangming District Center for Disease Control and Prevention |
| 116 | SPVP110002 | O1:KUT | 2011 | food | Guangming District Center for Disease Control and Prevention |
| 117 | SPVP110006 | OUT:KUT | 2011 | food | Guangming District Center for Disease Control and Prevention |
| 118 | SPVP110009 | O2:KUT | 2011 | food | Guangming District Center for Disease Control and Prevention |
| 119 | SPVP110012 | O8:KUT | 2011 | food | Futian District Center for Disease Control and Prevention |
| 120 | SPVP110014 | O3:KUT | 2011 | food | Futian District Center for Disease Control and Prevention |
| 121 | SPVP110015 | O4:KUT | 2011 | food | Futian District Center for Disease Control and Prevention |
| 122 | SPVP110021 | O3:KUT | 2011 | food | Baoan District Center for Disease Control and Prevention |
| 123 | SPVP110029 | O2:KUT | 2011 | food | Futian District Center for Disease Control and Prevention |
| 124 | SPVP110031 | O2:KUT | 2011 | food | Futian District Center for Disease Control and Prevention |
| 125 | SPVP110035 | O1:KUT | 2011 | food | Guangming District Center for Disease Control and Prevention |
| 126 | SPVP110038 | O3:KUT | 2011 | food | Futian District Center for Disease Control and Prevention |
| 127 | SPVP110044 | O1:KUT | 2011 | food | Guangming District Center for Disease Control and Prevention |
| 128 | SPVP110048 | O2:KUT | 2011 | food | Guangming District Center for Disease Control and Prevention |
| 129 | SPVP110050 | O3:KUT | 2011 | food | Shenzhen Center for Disease Control and Prevention |
| 130 | SPVP110051 | O1:KUT | 2011 | food | Shenzhen Center for Disease Control and Prevention |
| 131 | SPVP110052 | O11:KUT | 2011 | food | Shenzhen Center for Disease Control and Prevention |
| 132 | SPVP110053 | O2:KUT | 2011 | food | Shenzhen Center for Disease Control and Prevention |
| 133 | SPVP110060 | O2:KUT | 2011 | food | Nanshan District Center for Disease Control and Prevention |
| 134 | SPVP110062 | O2:KUT | 2011 | food | Nanshan District Center for Disease Control and Prevention |
| 135 | SPVP110063 | O2:KUT | 2011 | food | Nanshan District Center for Disease Control and Prevention |
| 136 | SPVP110086 | O3:KUT | 2011 | food | Longgang District Center for Disease Control and Prevention |
| 137 | SPVP110092 | O3:KUT | 2011 | food | Futian District Center for Disease Control and Prevention |
| 138 | SPVP110094 | O11:KUT | 2011 | food | Futian District Center for Disease Control and Prevention |
| 139 | SPVP110096 | O2:KUT | 2011 | food | Futian District Center for Disease Control and Prevention |
| 140 | SPVP110098 | O2:KUT | 2011 | food | Futian District Center for Disease Control and Prevention |
| 141 | VP11070 | O3:KUT | 2011 | clinical | Nanshan District Center for Disease Control and Prevention |
| 142 | VP11119 | O1:KUT | 2011 | clinical | Xixiang People's Hospital |
| 143 | VP11139 | O1:KUT | 2011 | clinical | Xixiang People's Hospital |
| 144 | VP11158 | O1:KUT | 2011 | clinical | Xixiang People's Hospital |
| 145 | VP11198 | O1:KUT | 2011 | clinical | Xixiang People's Hospital |
| 146 | VP11244 | O3:KUT | 2011 | clinical | Nanshan People's Hospital |
| 147 | VP11248 | O10:KUT | 2011 | clinical | Xixiang People's Hospital |
| 148 | VP11255 | O1:KUT | 2011 | food | Nanshan District Center for Disease Control and Prevention |
| 149 | VP11271 | O1:KUT | 2011 | clinical | Xixiang People's Hospital |
| 150 | VP11274 | O2:KUT | 2011 | clinical | Guangming People's Hospital |
| 151 | VP11310 | O1:KUT | 2011 | clinical | Nanshan People's Hospital |
| 152 | VP11312 | O1:KUT | 2011 | clinical | Xixiang People's Hospital |
| 153 | VP11314 | O1:KUT | 2011 | clinical | Xixiang People's Hospital |
| 154 | VP11315 | O1:KUT | 2011 | clinical | Shenzhen Hospital of Peking University |
| 155 | VP12004 | O1:KUT | 2012 | clinical | Xixiang People's Hospital |
| 156 | VP12014 | O10:KUT | 2012 | clinical | Nanshan People's Hospital |
| 157 | VP12015 | O1:KUT | 2012 | clinical | Nanshan District Center for Disease Control and Prevention |
| 158 | VP12023 | O1:KUT | 2012 | clinical | Longhua People's Hospital |
| 159 | VP12074 | O1:KUT | 2012 | clinical | Nanshan People's Hospital |
| 160 | VP12081 | OUT:KUT | 2012 | clinical | Longgang District Center for Disease Control and Prevention |
| 161 | VP12114 | O1:KUT | 2012 | clinical | Longhua People's Hospital |
| 162 | VP12122 | OUT:KUT | 2012 | clinical | Longgang District Center for Disease Control and Prevention |
| 163 | VP12145 | O1:KUT | 2012 | clinical | Xixiang People's Hospital |
| 164 | VP12208 | O1:KUT | 2012 | clinical | Nanshan People's Hospital |
| 165 | VP13005 | O4:KUT | 2013 | clinical | Xixiang People's Hospital |
| 166 | VP13007 | O4:KUT | 2013 | clinical | Xixiang People's Hospital |
| 167 | VP13009 | O1:KUT | 2013 | clinical | Shenzhen Hospital of Peking University |
| 168 | SPVP140164 | O10:KUT | 2014 | food | Shenzhen Center for Disease Control and Prevention |
| 169 | VP14031 | OUT:KUT | 2014 | food | Fuzhou Center for Disease Control and Prevention |
| 170 | VP14032 | OUT:KUT | 2014 | food | Fuzhou Center for Disease Control and Prevention |
| 171 | VP14033 | OUT:KUT | 2014 | food | Fuzhou Center for Disease Control and Prevention |
| 172 | VP14034 | OUT:KUT | 2014 | food | Fuzhou Center for Disease Control and Prevention |
| 173 | VP14035 | O10:KUT | 2014 | food | Fuzhou Center for Disease Control and Prevention |
| 174 | VP14036 | OUT:KUT | 2014 | food | Futian District Center for Disease Control and Prevention |
| 175 | VP14039 | OUT:KUT | 2014 | food | Fuzhou Center for Disease Control and Prevention |
| 176 | VP14041 | O3:KUT | 2014 | food | Shenzhen Center for Disease Control and Prevention |
| 177 | VP15006 | O3:KUT | 2015 | clinical | Longgang People's Hospital |
| 178 | VP15157 | O4:KUT | 2015 | clinical | Longhua People's Hospital |
| 179 | VP15200 | O1:KUT | 2015 | food | Yantian District Center for Disease Control and Prevention |
| 180 | VP15201 | O4:KUT | 2015 | clinical | Xixiang People's Hospital |
| 181 | VP15202 | O4:KUT | 2015 | clinical | Xixiang People's Hospital |
| 182 | VP16003 | O1:KUT | 2016 | clinical | Xixiang People's Hospital |
| 183 | VP16012 | O4:KUT | 2016 | clinical | Baoan Central Hospital |
| 184 | VP16019 | O4:KUT | 2016 | clinical | Longgang District Center for Disease Control and Prevention |
| 185 | VP16020 | O4:KUT | 2016 | clinical | Longgang District Center for Disease Control and Prevention |
| 186 | VP16021 | O4:KUT | 2016 | clinical | Longgang District Center for Disease Control and Prevention |
| 187 | VP16022 | O4:KUT | 2016 | clinical | Longgang District Center for Disease Control and Prevention |
| 188 | VP16041 | O4:KUT | 2016 | clinical | Longgang Central Hospital |
| 189 | VP16058 | O2:KUT | 2016 | clinical | Songgang People's Hospital |
| 190 | VP16065 | O4:KUT | 2016 | clinical | Baoan Central Hospital |
| 191 | VP16079 | O2:KUT | 2016 | clinical | Nanshan District Center for Disease Control and Prevention |
| 192 | VP16083 | O1:KUT | 2016 | clinical | Baoan Central Hospital |
| 193 | VP16084 | O4:KUT | 2016 | clinical | Baoan Central Hospital |
| 194 | VP16085 | O1:KUT | 2016 | clinical | Baoan Central Hospital |
| 195 | VP16086 | O4:KUT | 2016 | clinical | Songgang People's Hospital |
| 196 | VP16093 | O1:KUT | 2016 | clinical | Baoan Central Hospital |
| 197 | VP16096 | O4:KUT | 2016 | clinical | Baoan Central Hospital |
| 198 | VP16097 | O4:KUT | 2016 | clinical | Baoan Central Hospital |
| 199 | VP16165 | O1:KUT | 2016 | clinical | Longhua People's Hospital |
| 200 | VP16166 | O3:KUT | 2016 | clinical | Longhua People's Hospital |
| 201 | VP16175 | O1:KUT | 2016 | clinical | Xixiang People's Hospital |
| 202 | VP17003 | O1:KUT | 2017 | clinical | Baoan Central Hospital |
| 203 | VP17005 | O4:KUT | 2017 | clinical | Longgang People's Hospital |
| 204 | VP17030 | O4:KUT | 2017 | clinical | Longgang Central Hospital |
| 205 | VP17084 | O4:KUT | 2017 | clinical | Songgang People's Hospital |
| 206 | VP17106 | O3:KUT | 2017 | clinical | Baoan Central Hospital |
| 207 | VP17109 | O4:KUT | 2017 | clinical | Songgang People's Hospital |
| 208 | VP17127 | O1:KUT | 2017 | clinical | Shajing People's Hospital |
| 209 | VP17150 | O1:KUT | 2017 | clinical | Shenzhen Hospital of Peking University |
| 210 | VP17161 | O4:KUT | 2017 | clinical | Longhua People's Hospital |
| 211 | VP17165 | O4:KUT | 2017 | clinical | Songgang People's Hospital |
| 212 | VP18005 | O3:KUT | 2018 | clinical | Songgang People's Hospital |
| 213 | VP18008 | O4:KUT | 2018 | clinical | Longhua People's Hospital |
| 214 | VP18026 | O4:KUT | 2018 | clinical | Longgang People's Hospital |
| 215 | VP18042 | O11:KUT | 2018 | clinical | Nanshan People's Hospital |
| 216 | VP18045 | O4:KUT | 2018 | clinical | Longhua People's Hospital |
| 217 | VP18050 | O4:KUT | 2018 | clinical | Luohu People's Hospital |
| 218 | VP18051 | O11:KUT | 2018 | clinical | Luohu People's Hospital |
| 219 | VP18085 | OUT:KUT | 2018 | food | Nanshan District Center for Disease Control and Prevention |
| 220 | VP18086 | OUT:KUT | 2018 | food | Nanshan District Center for Disease Control and Prevention |
| 221 | VP18087 | OUT:KUT | 2018 | food | Nanshan District Center for Disease Control and Prevention |
| 222 | VP18103 | OUT:KUT | 2018 | clinical | Baoan Central Hospital |
| 223 | VP18115 | OUT:KUT | 2018 | clinical | Songgang People's Hospital |
| 224 | VP18129 | OUT:KUT | 2018 | clinical | Longhua People's Hospital |
| 225 | VP18130 | OUT:KUT | 2018 | clinical | Nanshan District Center for Disease Control and Prevention |
| 226 | VP18134 | O4:KUT | 2018 | clinical | Shenzhen Center for Disease Control and Prevention |
| 227 | VP18142 | O4:KUT | 2018 | clinical | Shenzhen Center for Disease Control and Prevention |
| 228 | VP18150 | O1:KUT | 2018 | clinical | Shenzhen Center for Disease Control and Prevention |
| 229 | VP18177 | O3:KUT | 2018 | clinical | Shenzhen Center for Disease Control and Prevention |
| 230 | VP18219 | O3:KUT | 2018 | clinical | Shenzhen Center for Disease Control and Prevention |
| 231 | VP18220 | O3:KUT | 2018 | clinical | Shenzhen Center for Disease Control and Prevention |
| 232 | VP18222 | O4:KUT | 2018 | clinical | Shenzhen Center for Disease Control and Prevention |
| 233 | VP18228 | O4:KUT | 2018 | clinical | Shenzhen Center for Disease Control and Prevention |
| 234 | VP18245 | O11:KUT | 2018 | clinical | Shenzhen Center for Disease Control and Prevention |
| 235 | VP18246 | O1:KUT | 2018 | clinical | Shenzhen Center for Disease Control and Prevention |
| 236 | VP18247 | O3:KUT | 2018 | clinical | Xixiang People's Hospital |

| **TABLE S4 \|** Universal primer and fluorogenic probes for the identification of *V. parahaemolyticus* K- serogroups in a three-tube system. | | |
| --- | --- | --- |
| **Name** | **Sequence(5′→3′)** |  |
| Universal primer-F | GTGGCAGGGCGCTACGAACAAT |  |
| Universal primer-R | GCCCAGCAAGATCCAATCTCA |  |
| ROX-probe | ROX-ACGACTCTGGCTGCTCGTTCGTGACG-BHQ2 |  |
| FAM-probe | FAM-TCGGTCCTTCATCGCTCAGCCTTCACCGG-BHQ1 |  |
| Cy5-probe | Cy5-CGGTGAGGCCCTTGGCAGGTTGCTATCACCC-BHQ2 |  |

| **TABLE S5 \|** Test results of 18 *V. parahaemolyticus* isolates by using the conventional serotyping, the MLMA assay and Sanger sequencing. | | | | |
| --- | --- | --- | --- | --- |
| **No.** | **Strain number** | **Conventional serotyping** | **MLMA** | **Sanger sequencing** |
| 1 | VP05239 | KUT | K6 | K6 |
| 2 | VP07050 | KUT | K19 | K19 |
| 3 | VP07194 | KUT | K17 | K17 |
| 4 | VP08085 | KUT | K17 | K17 |
| 5 | VP08218 | KUT | K6 | K6 |
| 6 | VP08223 | KUT | K6 | K6 |
| 7 | VP08293 | KUT | K17 | K17 |
| 8 | VP08302 | KUT | K32 | K32 |
| 9 | VP09003 | KUT | K32 | K32 |
| 10 | VP12014 | KUT | K32 | K32 |
| 11 | VP12122 | KUT | K68 | K68 |
| 12 | VP16175 | KUT | K29 | K29 |
| 13 | VP18247 | KUT | K6 | K6 |
| 14 | SPVP110031 | KUT | K17 | K17 |
| 15 | SPVP110044 | KUT | K19 | K19 |
| 16 | VP04079 | PolyvalentⅠ | K6 | K6 |
| 17 | VP08349 | PolyvalentⅠ | K6 | K6 |
| 18 | VP09451 | Polyvalent Ⅸ | K52 | K52 |

| **TABLE S6 \|** Comparison of MLMA assay and conventional serotyping tests in K-serogroups of *V. parahaemolyticus.* | | | | |
| --- | --- | --- | --- | --- |
| **No.** | **Serotypes** | **Number of isolates** | | **Inconsistent** |
|  |  | **MLMA** | **Conventional serotyping** |  |
| 1 | KUT | 218 | 218 | 0 |
| 2 | K6 | 75 | 69 | 6 |
| 3 | K56 | 55 | 55 | 0 |
| 4 | K29 | 42 | 41 | 1 |
| 5 | K8 | 39 | 39 | 0 |
| 6 | K36 | 28 | 28 | 0 |
| 7 | K68 | 28 | 27 | 1 |
| 8 | K3 | 22 | 22 | 0 |
| 9 | K25 | 19 | 19 | 0 |
| 10 | K9 | 15 | 15 | 0 |
| 11 | K18 | 10 | 10 | 0 |
| 12 | K13 | 8 | 8 | 0 |
| 13 | K17 | 8 | 4 | 4 |
| 14 | K32 | 6 | 3 | 3 |
| 15 | K19 | 3 | 1 | 2 |
| 16 | K42 | 3 | 3 | 0 |
| 17 | K28 | 2 | 2 | 0 |
| 18 | K34 | 2 | 2 | 0 |
| 19 | K5 | 1 | 1 | 0 |
| 20 | K12 | 1 | 1 | 0 |
| 21 | K20 | 1 | 1 | 0 |
| 22 | K24 | 1 | 1 | 0 |
| 23 | K30 | 1 | 1 | 0 |
| 24 | K37 | 1 | 1 | 0 |
| 25 | K41 | 1 | 1 | 0 |
| 26 | K48 | 1 | 1 | 0 |
| 27 | K52 | 1 | 0 | 1 |
| 28 | K54 | 1 | 1 | 0 |
| 29 | K67 | 1 | 1 | 0 |
| 30 | K70 | 1 | 1 | 0 |
| 31 | Total | 595 | 577 | 18 |

| **TABLE S7 \|** The strains information of *V.**parahaemolyticus* rare K- serotypes (n=5). | | | | | |
| --- | --- | --- | --- | --- | --- |
| **No.** | **Strain number** | **Serotypes** | **Source of strain** | **Virulence gene** | |
|  |  |  |  | ***tdh*** | ***trh*** |
| 1 | VP17151 | K54 | clinical isolate | **-** | **+** |
| 2 | VP15002 | K67 | clinical isolate | **-** | **-** |
| 3 | VP10023 | K24 | food isolate | **-** | **-** |
| 4 | VP09451 | K52 | food isolate | **-** | **-** |
| 5 | VP08109 | K70 | food isolate | **-** | **-** |

The data about 418 *V.parahaemolyticus* K-serogroups isolates that support the findings of this study have been deposited in the GenBank database (https://www.ncbi.nlm.nih.gov/). The assembly accession numbers and biosample numbers are listed in Table S8.

| **TABLE S8** \| The accession numbers and biosample numbers of 418 *V.parahaemolyticus* strains. | | | | | | | | |
| --- | --- | --- | --- | --- | --- | --- | --- | --- |
| **No.** | **Strain ID** | **Serotype** | **CPS gene cluster** | **Gene cluster length** | **ORF number** | **Year** | **Accession number** | **BioSample number** |
| 1 | VP1 | O4:K55 | YES | 37003 | 35 | 2008 | MT898026 | SAMN16783341 |
| 2 | VP104 | O1:K36 | YES | 33497 | 32 | 2010 | MT898100 | SAMN16783028 |
| 3 | VP107 | O4:K11 | YES | 35823 | 35 | 2007 | MT898391 | SAMN16783031 |
| 4 | VP109 | O4:K13 | YES | 34798 | 34 | 2008 | MT898004 | SAMN16783033 |
| 5 | VP113 | O9:K44 | YES | 42006 | 41 | 2008 | MT898206 | SAMN16783363 |
| 6 | VP132 | O1:K25 | YES | 36845 | 36 | 2008 | MT898283 | SAMN16783055 |
| 7 | VP135 | O4:K9 | YES | 39047 | 37 | 2008 | MT898341 | SAMN16783058 |
| 8 | VP16 | O10:K60 | YES | 43197 | 40 | 2015 | MT898076 | SAMN16783347 |
| 9 | VP161 | O4:K68 | YES | 44725 | 42 | 2010 | MT898029 | SAMN16783082 |
| 10 | VP179 | O4:K12 | YES | 41196 | 41 | 2012 | MT898299 | SAMN16783098 |
| 11 | VP187 | O4:K8 | YES | 39442 | 37 | 2011 | MT898178 | SAMN16783106 |
| 12 | VP190 | O5:K30 | YES | 32416 | 29 | 2008 | MT898163 | SAMN16783366 |
| 13 | VP192 | O11:K19 | YES | 37137 | 33 | 2007 | MT898099 | SAMN16783367 |
| 14 | VP195 | O3:K5 | YES | 30811 | 32 | 2008 | MT898262 | SAMN16783368 |
| 15 | VP197 | O6:K18 | YES | 32885 | 34 | 2015 | MT898213 | SAMN16783112 |
| 16 | VP198 | O4:K4 | YES | 37080 | 36 | 2015 | MT898208 | SAMN16783113 |
| 17 | VP199 | O1:K1 | YES | 37916 | 37 | 2009 | MT898145 | SAMN16783114 |
| 18 | VP200 | O1:K38 | YES | 28581 | 28 | 2008 | MT898150 | SAMN16783370 |
| 19 | VP202 | O1:K23 | YES | 36002 | 31 | 2008 | MT898072 | SAMN16783371 |
| 20 | VP203 | O1:K33 | YES | 32308 | 29 | 2008 | MT898408 | SAMN16783372 |
| 21 | VP204 | O3:K6 | YES | 33084 | 32 | 2017 | MT898107 | SAMN16783116 |
| 22 | VP205 | O3:K31 | YES | 34720 | 34 | 2008 | MT898311 | SAMN16783373 |
| 23 | VP206 | O11:K20 | YES | 29547 | 28 | 2008 | MT898261 | SAMN16783374 |
| 24 | VP229 | O3:K48 | YES | 34007 | 31 | 2008 | MT898401 | SAMN16783376 |
| 25 | VP230 | O3:K58 | YES | 33084 | 33 | 2012 | MT898195 | SAMN16783377 |
| 26 | VP234 | O8:K41 | YES | 32326 | 32 | 2010 | MT898360 | SAMN16783140 |
| 27 | VP238 | O4:K49 | YES | 39254 | 35 | 2010 | MT898369 | SAMN16783380 |
| 28 | VP239 | O3:K37 | YES | 27236 | 26 | 2009 | MT898231 | SAMN16783381 |
| 29 | VP245 | O2:K3 | YES | 64678 | 57 | 2008 | MT898350 | SAMN16783383 |
| 30 | VP247 | O3:K29 | YES | 31094 | 31 | 2008 | MT898031 | SAMN16783148 |
| 31 | VP32 | O10:K71 | YES | 31101 | 29 | 2012 | MT898269 | SAMN16783351 |
| 32 | VP321 | O4:K63 | YES | 43707 | 42 | 2008 | MT898183 | SAMN16783219 |
| 33 | VP33 | O1:K69 | YES | 36120 | 31 | 2011 | MT898042 | SAMN16783352 |
| 34 | VP334 | O1:K56 | YES | 29389 | 29 | 2016 | MT898411 | SAMN16783231 |
| 35 | VP4 | O4:K42 | YES | 35037 | 34 | 2008 | MT898215 | SAMN16783343 |
| 36 | VP43 | O5:K17 | YES | 57948 | 50 | 2009 | MT898343 | SAMN16782975 |
| 37 | VP439 | O8:K21 | YES | 29690 | 28 | 2009 | MT898297 | SAMN16783333 |
| 38 | VP53 | O2:K28 | YES | 67564 | 61 | 2010 | MT898376 | SAMN16782985 |
| 39 | VP6 | O4:K34 | YES | 38125 | 36 | 2008 | MT898188 | SAMN16783344 |
| 40 | VP99 | O1:K32 | YES | 46096 | 42 | 2010 | MT898113 | SAMN16783361 |
| 41 | VP10 | O4:K42 | YES | 35049 | 34 | 2009 | MT898352 | SAMN16782951 |
| 42 | VP101 | O4:K68 | YES | 44725 | 42 | 2010 | MT898361 | SAMN16783026 |
| 43 | VP102 | O1:K36 | YES | 33497 | 32 | 2011 | MT898147 | SAMN16783027 |
| 44 | VP103 | O1:K36 | YES | 33503 | 32 | 2010 | MT898402 | SAMN16783362 |
| 45 | VP106 | O4:K68 | YES | 44725 | 42 | 2007 | MT898324 | SAMN16783030 |
| 46 | VP108 | O4:K11 | YES | 35823 | 35 | 2007 | MT898365 | SAMN16783032 |
| 47 | VP11 | O4:K42 | YES | 35043 | 34 | 2009 | MT898035 | SAMN16782952 |
| 48 | VP110 | O4:K11 | YES | 35823 | 35 | 2010 | MT898255 | SAMN16783034 |
| 49 | VP111 | O1:K36 | YES | 33496 | 32 | 2012 | MT898118 | SAMN16783035 |
| 50 | VP112 | O4:K68 | YES | 44726 | 42 | 2011 | MT898128 | SAMN16783036 |
| 51 | VP114 | O9:K44 | YES | 42006 | 41 | 2008 | MT898123 | SAMN16783037 |
| 52 | VP115 | O9:K44 | YES | 42006 | 41 | 2008 | MT898225 | SAMN16783038 |
| 53 | VP116 | O9:K44 | YES | 42006 | 41 | 2008 | MT898134 | SAMN16783039 |
| 54 | VP117 | O9:K44 | YES | 42006 | 41 | 2008 | MT898170 | SAMN16783040 |
| 55 | VP118 | O9:K44 | YES | 42006 | 41 | 2008 | MT898169 | SAMN16783041 |
| 56 | VP119 | O9:K44 | YES | 42006 | 41 | 2008 | MT898148 | SAMN16783042 |
| 57 | VP120 | O9:K44 | YES | 42006 | 41 | 2008 | MT898083 | SAMN16783043 |
| 58 | VP121 | O1:K36 | YES | 33497 | 32 | 2009 | MT898120 | SAMN16783044 |
| 59 | VP122 | O11:K36 | YES | 39034 | 34 | 2012 | MT898202 | SAMN16783045 |
| 60 | VP123 | O1:K36 | YES | 33497 | 32 | 2009 | MT898053 | SAMN16783046 |
| 61 | VP124 | O1:K36 | YES | 33497 | 32 | 2009 | MT898009 | SAMN16783047 |
| 62 | VP125 | O1:K36 | YES | 33497 | 32 | 2010 | MT898007 | SAMN16783048 |
| 63 | VP126 | O1:K36 | YES | 33497 | 32 | 2010 | MT898191 | SAMN16783049 |
| 64 | VP128 | O2:K28 | YES | 67563 | 61 | 2010 | MT898103 | SAMN16783051 |
| 65 | VP129 | O1:K36 | YES | 33497 | 32 | 2010 | MT898104 | SAMN16783052 |
| 66 | VP130 | O4:K68 | YES | 44725 | 42 | 2008 | MT898286 | SAMN16783053 |
| 67 | VP131 | O4:K68 | YES | 44725 | 42 | 2008 | MT898267 | SAMN16783054 |
| 68 | VP133 | O1:K25 | YES | 36845 | 36 | 2008 | MT898271 | SAMN16783056 |
| 69 | VP134 | O4:K68 | YES | 44725 | 42 | 2008 | MT898204 | SAMN16783057 |
| 70 | VP136 | O4:K9 | YES | 39047 | 37 | 2008 | MT898355 | SAMN16783059 |
| 71 | VP137 | O1:K25 | YES | 36845 | 36 | 2008 | MT898386 | SAMN16783060 |
| 72 | VP138 | O4:K9 | YES | 39047 | 37 | 2008 | MT898084 | SAMN16783061 |
| 73 | VP139 | O4:K68 | YES | 44725 | 42 | 2008 | MT898327 | SAMN16783062 |
| 74 | VP14 | O4:K42 | YES | 35049 | 34 | 2010 | MT898207 | SAMN16782954 |
| 75 | VP140 | O1:K25 | YES | 36845 | 36 | 2009 | MT898357 | SAMN16783063 |
| 76 | VP141 | O1:K25 | YES | 36845 | 36 | 2009 | MT898117 | SAMN16783064 |
| 77 | VP143 | O4:K68 | YES | 44724 | 42 | 2009 | MT898013 | SAMN16783066 |
| 78 | VP144 | O4:K9 | YES | 39047 | 37 | 2009 | MT898040 | SAMN16783067 |
| 79 | VP145 | O4:K9 | YES | 39047 | 37 | 2009 | MT898010 | SAMN16783068 |
| 80 | VP146 | O1:K25 | YES | 36857 | 36 | 2012 | MT898251 | SAMN16783069 |
| 81 | VP147 | O1:K25 | YES | 36846 | 36 | 2009 | MT898415 | SAMN16783070 |
| 82 | VP148 | O4:K9 | YES | 39047 | 37 | 2009 | MT898325 | SAMN16783071 |
| 83 | VP149 | O4:K9 | YES | 39047 | 37 | 2009 | MT898229 | SAMN16783072 |
| 84 | VP15 | O4:K34 | YES | 38223 | 36 | 2011 | MT898112 | SAMN16782955 |
| 85 | VP150 | O4:K9 | YES | 39047 | 37 | 2009 | MT898198 | SAMN16783073 |
| 86 | VP151 | O4:K9 | YES | 39047 | 37 | 2009 | MT898284 | SAMN16783074 |
| 87 | VP152 | O4:K9 | YES | 39047 | 37 | 2009 | MT898075 | SAMN16783075 |
| 88 | VP154 | O4:K9 | YES | 39047 | 37 | 2010 | MT898173 | SAMN16783077 |
| 89 | VP155 | O1:K25 | YES | 36845 | 36 | 2010 | MT898383 | SAMN16783078 |
| 90 | VP158 | O4:K68 | YES | 44725 | 42 | 2010 | MT898264 | SAMN16783079 |
| 91 | VP162 | O4:K9 | YES | 39047 | 37 | 2012 | MT898347 | SAMN16783083 |
| 92 | VP164 | O4:K68 | YES | 44725 | 42 | 2010 | MT898270 | SAMN16783085 |
| 93 | VP167 | O4:K68 | YES | 44725 | 42 | 2010 | MT898390 | SAMN16783088 |
| 94 | VP168 | O1:K25 | YES | 36845 | 36 | 2010 | MT898366 | SAMN16783089 |
| 95 | VP169 | O4:K9 | YES | 39047 | 37 | 2012 | MT898405 | SAMN16783364 |
| 96 | VP17 | O8:K41 | YES | 32333 | 32 | 2007 | MT898085 | SAMN16782956 |
| 97 | VP170 | O4:K68 | YES | 44726 | 42 | 2012 | MT898308 | SAMN16783090 |
| 98 | VP171 | O4:K9 | YES | 39035 | 37 | 2012 | MT898345 | SAMN16783091 |
| 99 | VP172 | O4:K9 | YES | 39047 | 37 | 2012 | MT898417 | SAMN16783092 |
| 100 | VP173 | O4:K9 | YES | 39047 | 37 | 2012 | MT898164 | SAMN16783093 |
| 101 | VP174 | O4:K9 | YES | 39047 | 37 | 2012 | MT898373 | SAMN16783094 |
| 102 | VP175 | O4:K9 | YES | 39047 | 37 | 2011 | MT898379 | SAMN16783095 |
| 103 | VP176 | O4:K68 | YES | 44724 | 42 | 2011 | MT898339 | SAMN16783096 |
| 104 | VP177 | O1:K25 | YES | 39329 | 38 | 2007 | MT898233 | SAMN16783365 |
| 105 | VP178 | O4:K68 | YES | 44724 | 42 | 2011 | MT898309 | SAMN16783097 |
| 106 | VP180 | O1:K55 | YES | 37003 | 35 | 2016 | MT898130 | SAMN16783099 |
| 107 | VP181 | O1:K25 | YES | 36845 | 36 | 2007 | MT898342 | SAMN16783100 |
| 108 | VP182 | O1:K25 | YES | 36846 | 36 | 2007 | MT898218 | SAMN16783101 |
| 109 | VP183 | O1:K25 | YES | 36845 | 36 | 2007 | MT898258 | SAMN16783102 |
| 110 | VP184 | O4:K68 | YES | 44725 | 42 | 2007 | MT898371 | SAMN16783103 |
| 111 | VP185 | O4:K68 | YES | 44724 | 42 | 2011 | MT898079 | SAMN16783104 |
| 112 | VP186 | O1:K25 | YES | 36845 | 36 | 2011 | MT898414 | SAMN16783105 |
| 113 | VP188 | O3:K6 | YES | 33084 | 32 | 2012 | MT898094 | SAMN16783107 |
| 114 | VP19 | O4:K55 | YES | 37003 | 35 | 2012 | MT898273 | SAMN16782958 |
| 115 | VP191 | O5:K30 | YES | 32407 | 30 | 2008 | MT898392 | SAMN16783109 |
| 116 | VP193 | OUT:K28 | YES | 67560 | 61 | 2014 | MT898372 | SAMN16783110 |
| 117 | VP194 | O4:K68 | YES | 44726 | 42 | 2015 | MT898092 | SAMN16783111 |
| 118 | VP196 | O3:K37 | YES | 36438 | 36 | 2009 | MT898250 | SAMN16783369 |
| 119 | VP2 | O4:K55 | YES | 37003 | 35 | 2008 | MT898293 | SAMN16782947 |
| 120 | VP20 | O8:K41 | YES | 32325 | 32 | 2012 | MT898020 | SAMN16782959 |
| 121 | VP201 | O4:K33 | YES | 32306 | 30 | 2008 | MT898023 | SAMN16783115 |
| 122 | VP207 | O11:K20 | YES | 29567 | 28 | 2010 | MT898189 | SAMN16783117 |
| 123 | VP208 | O10:K60 | YES | 43197 | 40 | 2015 | MT898194 | SAMN16783375 |
| 124 | VP209 | O1:K36 | YES | 33497 | 32 | 2016 | MT898294 | SAMN16783118 |
| 125 | VP21 | O8:K41 | YES | 32319 | 32 | 2012 | MT898185 | SAMN16782960 |
| 126 | VP210 | O1:K36 | YES | 33497 | 32 | 2008 | MT898190 | SAMN16783119 |
| 127 | VP211 | O1:K36 | YES | 33497 | 32 | 2007 | MT898080 | SAMN16783120 |
| 128 | VP212 | O1:K36 | YES | 33497 | 32 | 2009 | MT898039 | SAMN16783121 |
| 129 | VP213 | O1:K36 | YES | 33497 | 32 | 2007 | MT898179 | SAMN16783122 |
| 130 | VP214 | O1:K36 | YES | 33497 | 32 | 2009 | MT898168 | SAMN16783123 |
| 131 | VP215 | O1:K36 | YES | 33497 | 32 | 2010 | MT898002 | SAMN16783124 |
| 132 | VP216 | O1:K36 | YES | 33497 | 32 | 2010 | MT898237 | SAMN16783125 |
| 133 | VP217 | O1:K36 | YES | 33497 | 32 | 2010 | MT898110 | SAMN16783126 |
| 134 | VP218 | O1:K36 | YES | 33497 | 32 | 2010 | MT898090 | SAMN16783127 |
| 135 | VP219 | O1:K36 | YES | 33497 | 32 | 2010 | MT898331 | SAMN16783128 |
| 136 | VP220 | O1:K36 | YES | 33497 | 32 | 2010 | MT898006 | SAMN16783129 |
| 137 | VP221 | O1:K36 | YES | 33497 | 32 | 2010 | MT898165 | SAMN16783130 |
| 138 | VP222 | O1:K36 | YES | 33497 | 32 | 2010 | MT898081 | SAMN16783131 |
| 139 | VP223 | O1:K36 | YES | 33497 | 32 | 2010 | MT898091 | SAMN16783132 |
| 140 | VP224 | O1:K36 | YES | 33497 | 32 | 2010 | MT898254 | SAMN16783133 |
| 141 | VP225 | O1:K36 | YES | 33497 | 32 | 2010 | MT898197 | SAMN16783134 |
| 142 | VP226 | O1:K36 | YES | 33497 | 32 | 2010 | MT898143 | SAMN16783135 |
| 143 | VP228 | O1:K36 | YES | 33497 | 32 | 2010 | MT898337 | SAMN16783137 |
| 144 | VP23 | O10:K60 | YES | 43197 | 40 | 2015 | MT898157 | SAMN16783348 |
| 145 | VP231 | OUT:K17 | YES | 57941 | 50 | 2015 | MT898332 | SAMN16783138 |
| 146 | VP232 | O1:K25 | YES | 36846 | 36 | 2008 | MT898116 | SAMN16783139 |
| 147 | VP233 | O1:K56 | YES | 29398 | 29 | 2008 | MT898109 | SAMN16783378 |
| 148 | VP235 | O4:K68 | YES | 44725 | 42 | 2007 | MT898119 | SAMN16783379 |
| 149 | VP237 | O4:K68 | YES | 44724 | 42 | 2009 | MT898121 | SAMN16783142 |
| 150 | VP24 | O10:K60 | YES | 43197 | 40 | 2015 | MT898322 | SAMN16783349 |
| 151 | VP244 | O4:K11 | YES | 35823 | 35 | 2016 | MT898377 | SAMN16783146 |
| 152 | VP246 | O2:K3 | YES | 64674 | 57 | 2008 | MT898263 | SAMN16783147 |
| 153 | VP248 | O2:K3 | YES | 64678 | 57 | 2008 | MT898292 | SAMN16783149 |
| 154 | VP249 | O3:K29 | YES | 31099 | 31 | 2008 | MT898290 | SAMN16783150 |
| 155 | VP25 | O4:K34 | YES | 38124 | 36 | 2015 | MT898210 | SAMN16782962 |
| 156 | VP250 | O3:K29 | YES | 31089 | 31 | 2009 | MT898221 | SAMN16783151 |
| 157 | VP251 | O3:K29 | YES | 31099 | 31 | 2009 | MT898180 | SAMN16783152 |
| 158 | VP252 | O2:K3 | YES | 64678 | 57 | 2010 | MT898223 | SAMN16783153 |
| 159 | VP253 | O2:K3 | YES | 64684 | 57 | 2009 | MT898217 | SAMN16783154 |
| 160 | VP254 | O2:K3 | YES | 64674 | 57 | 2009 | MT898115 | SAMN16783155 |
| 161 | VP255 | O3:K29 | YES | 31094 | 31 | 2010 | MT898410 | SAMN16783156 |
| 162 | VP256 | O3:K3 | YES | 64678 | 57 | 2011 | MT898059 | SAMN16783157 |
| 163 | VP258 | O2:K3 | YES | 64678 | 57 | 2009 | MT898385 | SAMN16783159 |
| 164 | VP259 | O3:K29 | YES | 31089 | 31 | 2009 | MT898018 | SAMN16783160 |
| 165 | VP260 | O2:K3 | YES | 64674 | 57 | 2011 | MT898407 | SAMN16783161 |
| 166 | VP263 | O2:K3 | YES | 64678 | 57 | 2010 | MT898028 | SAMN16783164 |
| 167 | VP265 | O2:K3 | YES | 64678 | 57 | 2013 | MT898257 | SAMN16783166 |
| 168 | VP266 | O3:K29 | YES | 31099 | 31 | 2007 | MT898082 | SAMN16783167 |
| 169 | VP267 | O3:K3 | YES | 64678 | 57 | 2007 | MT898419 | SAMN16783168 |
| 170 | VP268 | O3:K29 | YES | 31099 | 31 | 2012 | MT898330 | SAMN16783169 |
| 171 | VP27 | O4:K28 | YES | 67561 | 61 | 2016 | MT898032 | SAMN16782964 |
| 172 | VP272 | O3:K6 | YES | 33084 | 32 | 2017 | MT898124 | SAMN16783173 |
| 173 | VP273 | O4:K8 | YES | 39442 | 37 | 2007 | MT898228 | SAMN16783174 |
| 174 | VP274 | O4:K8 | YES | 39442 | 37 | 2007 | MT898129 | SAMN16783175 |
| 175 | VP275 | O4:K8 | YES | 39442 | 37 | 2008 | MT898326 | SAMN16783176 |
| 176 | VP276 | O1:K56 | YES | 29398 | 29 | 2008 | MT898328 | SAMN16783177 |
| 177 | VP277 | O4:K8 | YES | 39444 | 36 | 2008 | MT898378 | SAMN16783178 |
| 178 | VP278 | O1:K56 | YES | 29393 | 29 | 2008 | MT898362 | SAMN16783179 |
| 179 | VP279 | O4:K8 | YES | 39442 | 37 | 2008 | MT898149 | SAMN16783180 |
| 180 | VP28 | O4:K60 | YES | 43197 | 40 | 2009 | MT898253 | SAMN16782965 |
| 181 | VP280 | O4:K56 | YES | 29398 | 29 | 2008 | MT898412 | SAMN16783181 |
| 182 | VP281 | O1:K56 | YES | 29398 | 29 | 2008 | MT898260 | SAMN16783182 |
| 183 | VP282 | O1:K56 | YES | 29398 | 29 | 2008 | MT898077 | SAMN16783183 |
| 184 | VP283 | O4:K8 | YES | 39442 | 37 | 2009 | MT898021 | SAMN16783184 |
| 185 | VP284 | O4:K8 | YES | 39442 | 37 | 2009 | MT898275 | SAMN16783185 |
| 186 | VP286 | O4:K8 | YES | 39442 | 37 | 2009 | MT898382 | SAMN16783187 |
| 187 | VP288 | O4:K8 | YES | 39442 | 37 | 2010 | MT898289 | SAMN16783189 |
| 188 | VP289 | O1:K56 | YES | 29392 | 29 | 2010 | MT898316 | SAMN16783190 |
| 189 | VP29 | O1:K56 | YES | 29389 | 29 | 2015 | MT898019 | SAMN16782966 |
| 190 | VP290 | O1:K56 | YES | 29392 | 29 | 2010 | MT898232 | SAMN16783191 |
| 191 | VP291 | O1:K56 | YES | 29392 | 29 | 2010 | MT898043 | SAMN16783192 |
| 192 | VP292 | O1:K56 | YES | 29392 | 29 | 2010 | MT898192 | SAMN16783193 |
| 193 | VP293 | O4:K8 | YES | 39442 | 37 | 2010 | MT898172 | SAMN16783194 |
| 194 | VP294 | O1:K56 | YES | 29392 | 29 | 2010 | MT898068 | SAMN16783195 |
| 195 | VP295 | O1:K56 | YES | 29392 | 29 | 2010 | MT898146 | SAMN16783196 |
| 196 | VP296 | O4:K8 | YES | 39442 | 37 | 2010 | MT898181 | SAMN16783197 |
| 197 | VP297 | O1:K56 | YES | 29392 | 29 | 2010 | MT898236 | SAMN16783198 |
| 198 | VP298 | O4:K8 | YES | 39442 | 37 | 2016 | MT898186 | SAMN16783199 |
| 199 | VP299 | O1:K56 | YES | 29389 | 29 | 2012 | MT898418 | SAMN16783200 |
| 200 | VP3 | O4:K42 | YES | 35037 | 34 | 2008 | MT898321 | SAMN16783342 |
| 201 | VP30 | O2:K28 | YES | 67562 | 61 | 2010 | MT898370 | SAMN16783350 |
| 202 | VP302 | O3:K6 | YES | 33077 | 32 | 2015 | MT898105 | SAMN16783203 |
| 203 | VP304 | O8:K41 | YES | 32326 | 32 | 2008 | MT898140 | SAMN16783384 |
| 204 | VP305 | O1:K1 | YES | 37916 | 37 | 2008 | MT898302 | SAMN16783385 |
| 205 | VP307 | O1:K32 | YES | 38899 | 36 | 2009 | MT898344 | SAMN16783206 |
| 206 | VP309 | O8:K41 | YES | 32309 | 32 | 2009 | MT898266 | SAMN16783208 |
| 207 | VP31 | O2:K28 | YES | 67562 | 61 | 2010 | MT898363 | SAMN16782967 |
| 208 | VP310 | O1:K25 | YES | 36853 | 36 | 2010 | MT898313 | SAMN16783209 |
| 209 | VP311 | O8:K41 | YES | 32322 | 31 | 2010 | MT898065 | SAMN16783210 |
| 210 | VP314 | O1:K32 | YES | 46396 | 42 | 2010 | MT898064 | SAMN16783213 |
| 211 | VP317 | O4:K4 | YES | 37080 | 36 | 2010 | MT898368 | SAMN16783216 |
| 212 | VP319 | O4:K4 | YES | 37080 | 36 | 2007 | MT898152 | SAMN16783386 |
| 213 | VP320 | O8:K41 | YES | 32326 | 32 | 2007 | MT898298 | SAMN16783218 |
| 214 | VP322 | O1:K1 | YES | 37916 | 37 | 2008 | MT898306 | SAMN16783220 |
| 215 | VP323 | O10:K19 | YES | 39307 | 36 | 2010 | MT898122 | SAMN16783221 |
| 216 | VP324 | O3:K6 | YES | 33084 | 32 | 2015 | MT898396 | SAMN16783222 |
| 217 | VP325 | O3:K8 | YES | 39442 | 37 | 2012 | MT898205 | SAMN16783223 |
| 218 | VP326 | O8:K8 | YES | 39442 | 37 | 2012 | MT898177 | SAMN16783224 |
| 219 | VP327 | O3:K56 | YES | 29389 | 29 | 2012 | MT898212 | SAMN16783225 |
| 220 | VP328 | O1:K8 | YES | 39442 | 37 | 2007 | MT898384 | SAMN16783226 |
| 221 | VP329 | OUT:K56 | YES | 29389 | 29 | 2013 | MT898153 | SAMN16783227 |
| 222 | VP330 | O1:K56 | YES | 29399 | 29 | 2010 | MT898317 | SAMN16783228 |
| 223 | VP331 | O4:K8 | YES | 39442 | 37 | 2015 | MT898387 | SAMN16783387 |
| 224 | VP332 | O1:K56 | YES | 29389 | 29 | 2015 | MT898063 | SAMN16783229 |
| 225 | VP333 | O4:K8 | YES | 39442 | 37 | 2015 | MT898216 | SAMN16783230 |
| 226 | VP335 | O1:K56 | YES | 29389 | 29 | 2016 | MT898086 | SAMN16783232 |
| 227 | VP336 | O4:K8 | YES | 39442 | 37 | 2011 | MT898158 | SAMN16783233 |
| 228 | VP337 | O4:K8 | YES | 39442 | 37 | 2011 | MT898167 | SAMN16783234 |
| 229 | VP338 | O4:K8 | YES | 39444 | 36 | 2012 | MT898022 | SAMN16783235 |
| 230 | VP339 | O4:K8 | YES | 39442 | 37 | 2012 | MT898155 | SAMN16783236 |
| 231 | VP34 | O4:K63 | YES | 43706 | 41 | 2008 | MT898012 | SAMN16782968 |
| 232 | VP340 | O4:K8 | YES | 39442 | 37 | 2012 | MT898312 | SAMN16783237 |
| 233 | VP341 | O3:K6 | YES | 33084 | 32 | 2017 | MT898219 | SAMN16783238 |
| 234 | VP342 | O3:K6 | YES | 33084 | 32 | 2017 | MT898323 | SAMN16783239 |
| 235 | VP343 | O3:K6 | YES | 33084 | 32 | 2017 | MT898102 | SAMN16783240 |
| 236 | VP345 | O4:K8 | YES | 39442 | 37 | 2013 | MT898127 | SAMN16783242 |
| 237 | VP346 | O4:K8 | YES | 39442 | 37 | 2013 | MT898154 | SAMN16783243 |
| 238 | VP347 | O1:K56 | YES | 29389 | 29 | 2013 | MT898175 | SAMN16783244 |
| 239 | VP348 | O4:K8 | YES | 39442 | 37 | 2017 | MT898319 | SAMN16783245 |
| 240 | VP349 | O4:K25 | YES | 36845 | 36 | 2016 | MT898394 | SAMN16783246 |
| 241 | VP35 | O4:K63 | YES | 43590 | 40 | 2008 | MT898226 | SAMN16783353 |
| 242 | VP353 | O5:K17 | YES | 57950 | 50 | 2008 | MT898142 | SAMN16783249 |
| 243 | VP354 | O3:K6 | YES | 33097 | 32 | 2008 | MT898310 | SAMN16783250 |
| 244 | VP357 | O3:K6 | YES | 33084 | 32 | 2008 | MT898380 | SAMN16783253 |
| 245 | VP359 | OUT:K12 | YES | 41289 | 40 | 2013 | MT898278 | SAMN16783255 |
| 246 | VP36 | O4:K63 | YES | 43708 | 43 | 2010 | MT898108 | SAMN16782969 |
| 247 | VP363 | O3:K6 | YES | 33084 | 32 | 2015 | MT898048 | SAMN16783259 |
| 248 | VP369 | O3:K6 | YES | 33084 | 32 | 2009 | MT898144 | SAMN16783265 |
| 249 | VP37 | O2:K28 | YES | 67562 | 61 | 2010 | MT898139 | SAMN16782970 |
| 250 | VP372 | O3:K6 | YES | 33084 | 32 | 2010 | MT898234 | SAMN16783268 |
| 251 | VP373 | O3:K6 | YES | 33084 | 32 | 2010 | MT898096 | SAMN16783269 |
| 252 | VP375 | OUT:K33 | YES | 32305 | 29 | 2010 | MT898196 | SAMN16783271 |
| 253 | VP377 | O4:K8 | YES | 39442 | 37 | 2015 | MT898304 | SAMN16783273 |
| 254 | VP378 | O3:K29 | YES | 31094 | 31 | 2012 | MT898397 | SAMN16783388 |
| 255 | VP38 | O5:K17 | YES | 53956 | 47 | 2008 | MT898025 | SAMN16782971 |
| 256 | VP380 | O4:K8 | YES | 39442 | 37 | 2012 | MT898069 | SAMN16783275 |
| 257 | VP381 | OUT:K56 | YES | 29398 | 29 | 2009 | MT898354 | SAMN16783276 |
| 258 | VP384 | O3:K6 | YES | 33084 | 32 | 2013 | MT898052 | SAMN16783279 |
| 259 | VP390 | O3:K6 | YES | 33084 | 32 | 2011 | MT898246 | SAMN16783285 |
| 260 | VP391 | O3:K6 | YES | 33084 | 32 | 2007 | MT898141 | SAMN16783286 |
| 261 | VP392 | O3:K6 | YES | 33084 | 32 | 2007 | MT898252 | SAMN16783287 |
| 262 | VP394 | O3:K6 | YES | 33084 | 32 | 2008 | MT898348 | SAMN16783289 |
| 263 | VP396 | O3:K6 | YES | 33091 | 32 | 2016 | MT898135 | SAMN16783291 |
| 264 | VP397 | O3:K29 | YES | 31094 | 31 | 2011 | MT898187 | SAMN16783292 |
| 265 | VP398 | O3:K6 | YES | 33084 | 32 | 2016 | MT898174 | SAMN16783293 |
| 266 | VP399 | O1:K36 | YES | 33497 | 32 | 2016 | MT898067 | SAMN16783294 |
| 267 | VP40 | O2:K3 | YES | 64678 | 57 | 2010 | MT898235 | SAMN16782973 |
| 268 | VP401 | O3:K6 | YES | 33084 | 32 | 2012 | MT898016 | SAMN16783296 |
| 269 | VP402 | O3:K6 | YES | 33084 | 32 | 2012 | MT898193 | SAMN16783297 |
| 270 | VP404 | O3:K6 | YES | 33084 | 32 | 2012 | MT898244 | SAMN16783299 |
| 271 | VP405 | O3:K6 | YES | 33077 | 32 | 2017 | MT898066 | SAMN16783300 |
| 272 | VP406 | O4:K42 | YES | 35049 | 34 | 2011 | MT898056 | SAMN16783301 |
| 273 | VP407 | O10:K60 | YES | 43197 | 40 | 2017 | MT898074 | SAMN16783389 |
| 274 | VP41 | O2:K28 | YES | 67562 | 61 | 2008 | MT898078 | SAMN16782974 |
| 275 | VP416 | O1:K56 | YES | 29389 | 29 | 2017 | MT898070 | SAMN16783310 |
| 276 | VP42 | O5:K17 | YES | 57950 | 50 | 2009 | MT898151 | SAMN16783354 |
| 277 | VP421 | OUT:K34 | YES | 38125 | 36 | 2017 | MT898133 | SAMN16783315 |
| 278 | VP428 | O4:K8 | YES | 39442 | 37 | 2017 | MT898303 | SAMN16783322 |
| 279 | VP434 | O11:K19 | YES | 37137 | 33 | 2007 | MT898279 | SAMN16783328 |
| 280 | VP44 | O5:K17 | YES | 53582 | 47 | 2010 | MT898176 | SAMN16782976 |
| 281 | VP440 | O3:K6 | YES | 33084 | 32 | 2017 | MT898238 | SAMN16783334 |
| 282 | VP441 | O3:K6 | YES | 33084 | 32 | 2017 | MT898015 | SAMN16783335 |
| 283 | VP442 | O4:K8 | YES | 39448 | 37 | 2017 | MT898398 | SAMN16783336 |
| 284 | VP443 | O1:K36 | YES | 33504 | 33 | 2017 | MT898265 | SAMN16783337 |
| 285 | VP444 | O1:K25 | YES | 36848 | 36 | 2017 | MT898093 | SAMN16783338 |
| 286 | VP45 | O1:K36 | YES | 26539 | 26 | 2010 | MT898138 | SAMN16782977 |
| 287 | VP48 | OUT:K32 | YES | 46397 | 43 | 2010 | MT898126 | SAMN16782980 |
| 288 | VP49 | O2:K28 | YES | 67561 | 61 | 2010 | MT898011 | SAMN16782981 |
| 289 | VP50 | O5:K17 | YES | 53580 | 47 | 2010 | MT898296 | SAMN16782982 |
| 290 | VP51 | O5:K17 | YES | 57948 | 50 | 2010 | MT898272 | SAMN16782983 |
| 291 | VP54 | O2:K28 | YES | 67560 | 60 | 2010 | MT898041 | SAMN16782986 |
| 292 | VP55 | O2:K28 | YES | 67560 | 60 | 2010 | MT898329 | SAMN16782987 |
| 293 | VP56 | O2:K28 | YES | 67571 | 62 | 2010 | MT898211 | SAMN16782988 |
| 294 | VP57 | O5:K17 | YES | 52053 | 45 | 2010 | MT898295 | SAMN16782989 |
| 295 | VP58 | O5:K17 | YES | 52053 | 45 | 2010 | MT898201 | SAMN16782990 |
| 296 | VP59 | O4:K12 | YES | 41200 | 41 | 2007 | MT898288 | SAMN16783355 |
| 297 | VP60 | O5:K17 | YES | 56454 | 48 | 2011 | MT898182 | SAMN16782991 |
| 298 | VP61 | O5:K17 | YES | 53577 | 47 | 2011 | MT898058 | SAMN16782992 |
| 299 | VP63 | O3:K6 | YES | 33084 | 32 | 2011 | MT898054 | SAMN16782994 |
| 300 | VP64 | O4:K12 | YES | 41196 | 41 | 2012 | MT898301 | SAMN16782995 |
| 301 | VP65 | O10:K60 | YES | 43197 | 40 | 2013 | MT898243 | SAMN16783356 |
| 302 | VP66 | O5:K17 | YES | 53579 | 47 | 2013 | MT898336 | SAMN16782996 |
| 303 | VP67 | O6:K18 | YES | 32885 | 34 | 2013 | MT898060 | SAMN16783357 |
| 304 | VP69 | O4:K12 | YES | 41220 | 41 | 2014 | MT898003 | SAMN16782998 |
| 305 | VP7 | O4:K42 | YES | 35047 | 34 | 2008 | MT898404 | SAMN16782949 |
| 306 | VP70 | O2:K28 | YES | 67560 | 61 | 2007 | MT898356 | SAMN16782999 |
| 307 | VP71 | O6:K18 | YES | 32895 | 33 | 2009 | MT898241 | SAMN16783000 |
| 308 | VP72 | O10:K60 | YES | 43197 | 40 | 2017 | MT898393 | SAMN16783358 |
| 309 | VP73 | O4:K68 | YES | 44725 | 42 | 2007 | MT898024 | SAMN16783001 |
| 310 | VP74 | O4:K13 | YES | 34798 | 34 | 2008 | MT898305 | SAMN16783002 |
| 311 | VP75 | O4:K13 | YES | 34798 | 34 | 2008 | MT898227 | SAMN16783003 |
| 312 | VP76 | O4:K13 | YES | 34798 | 34 | 2008 | MT898161 | SAMN16783004 |
| 313 | VP77 | O4:K13 | YES | 34798 | 34 | 2008 | MT898291 | SAMN16783005 |
| 314 | VP78 | O4:K13 | YES | 34798 | 34 | 2008 | MT898184 | SAMN16783006 |
| 315 | VP79 | O4:K25 | YES | 36845 | 36 | 2008 | MT898395 | SAMN16783007 |
| 316 | VP8 | O10:K60 | YES | 43197 | 40 | 2016 | MT898240 | SAMN16783345 |
| 317 | VP80 | O4:K13 | YES | 34798 | 34 | 2008 | MT898005 | SAMN16783008 |
| 318 | VP81 | O4:K11 | YES | 35823 | 35 | 2009 | MT898358 | SAMN16783009 |
| 319 | VP82 | O4:K13 | YES | 34798 | 34 | 2009 | MT898036 | SAMN16783010 |
| 320 | VP83 | O4:K13 | YES | 36101 | 35 | 2009 | MT898403 | SAMN16783011 |
| 321 | VP84 | O4:K68 | YES | 44724 | 42 | 2009 | MT898314 | SAMN16783012 |
| 322 | VP85 | O3:K6 | YES | 33084 | 32 | 2009 | MT898346 | SAMN16783013 |
| 323 | VP86 | O4:K11 | YES | 35817 | 35 | 2009 | MT898101 | SAMN16783014 |
| 324 | VP88 | O4:K68 | YES | 44726 | 42 | 2009 | MT898132 | SAMN16783016 |
| 325 | VP9 | O4:K42 | YES | 35047 | 34 | 2009 | MT898097 | SAMN16782950 |
| 326 | VP92 | O4:K68 | YES | 44726 | 42 | 2009 | MT898389 | SAMN16783020 |
| 327 | VP93 | O4:K68 | YES | 44726 | 42 | 2009 | MT898037 | SAMN16783021 |
| 328 | VP94 | O4:K13 | YES | 34798 | 34 | 2009 | MT898400 | SAMN16783359 |
| 329 | VP97 | O4:K11 | YES | 35817 | 35 | 2009 | MT898047 | SAMN16783360 |
| 330 | VP98 | O4:K13 | YES | 34795 | 34 | 2009 | MT898136 | SAMN16783024 |
| 331 | VP12 | O8:KUT | YES | 46407 | 42 | 2009 | MT898073 | SAMN16783346 |
| 332 | VP127 | O5:KUT | YES | 36604 | 34 | 2009 | MT898409 | SAMN16783050 |
| 333 | VP13 | O8:KUT | YES | 32221 | 29 | 2009 | MT898030 | SAMN16782953 |
| 334 | VP153 | O10:KUT | YES | 40420 | 35 | 2010 | MT898008 | SAMN16783076 |
| 335 | VP18 | OUT:KUT | YES | 33065 | 33 | 2007 | MT898014 | SAMN16782957 |
| 336 | VP189 | O8:KUT | YES | 35286 | 32 | 2008 | MT898027 | SAMN16783108 |
| 337 | VP22 | OUT:KUT | YES | 33090 | 32 | 2008 | MT898062 | SAMN16782961 |
| 338 | VP236 | O6:KUT | YES | 37868 | 38 | 2009 | MT898307 | SAMN16783141 |
| 339 | VP240 | O2:KUT | YES | 66567 | 59 | 2009 | MT898057 | SAMN16783143 |
| 340 | VP241 | O11:KUT | YES | 50375 | 44 | 2009 | MT898203 | SAMN16783144 |
| 341 | VP257 | O4:KUT | YES | 35133 | 32 | 2009 | MT898224 | SAMN16783158 |
| 342 | VP262 | O4:KUT | YES | 35133 | 33 | 2016 | MT898351 | SAMN16783163 |
| 343 | VP264 | O3:KUT | YES | 33084 | 32 | 2010 | MT898038 | SAMN16783165 |
| 344 | VP269 | O2:KUT | YES | 65701 | 61 | 2012 | MT898364 | SAMN16783170 |
| 345 | VP271 | O2:KUT | YES | 78621 | 68 | 2010 | MT898199 | SAMN16783172 |
| 346 | VP285 | O4:KUT | YES | 33084 | 32 | 2015 | MT898095 | SAMN16783186 |
| 347 | VP287 | O4:KUT | YES | 35966 | 34 | 2010 | MT898214 | SAMN16783188 |
| 348 | VP300 | O3:KUT | YES | 33524 | 32 | 2008 | MT898367 | SAMN16783201 |
| 349 | VP301 | O10:KUT | YES | 43966 | 39 | 2008 | MT898051 | SAMN16783202 |
| 350 | VP303 | O8:KUT | YES | 33540 | 29 | 2008 | MT898156 | SAMN16783204 |
| 351 | VP306 | O3:KUT | YES | 32297 | 31 | 2009 | MT898285 | SAMN16783205 |
| 352 | VP308 | O10:KUT | YES | 43498 | 41 | 2009 | MT898277 | SAMN16783207 |
| 353 | VP313 | O1:KUT | YES | 37632 | 32 | 2010 | MT898222 | SAMN16783212 |
| 354 | VP315 | O4:KUT | YES | 35094 | 32 | 2008 | MT898114 | SAMN16783214 |
| 355 | VP318 | OUT:KUT | YES | 32662 | 29 | 2010 | MT898300 | SAMN16783217 |
| 356 | VP344 | O4:KUT | YES | 39074 | 36 | 2016 | MT898159 | SAMN16783241 |
| 357 | VP351 | O1:KUT | YES | 30085 | 29 | 2016 | MT898281 | SAMN16783247 |
| 358 | VP352 | O4:KUT | YES | 35815 | 34 | 2008 | MT898282 | SAMN16783248 |
| 359 | VP355 | OUT:KUT | YES | 32880 | 32 | 2013 | MT898160 | SAMN16783251 |
| 360 | VP356 | O10:KUT | YES | 45518 | 42 | 2008 | MT898044 | SAMN16783252 |
| 361 | VP358 | OUT:KUT | YES | 39347 | 39 | 2009 | MT898274 | SAMN16783254 |
| 362 | VP360 | O11:KUT | YES | 36975 | 35 | 2014 | MT898046 | SAMN16783256 |
| 363 | VP361 | O1:KUT | YES | 37905 | 35 | 2009 | MT898166 | SAMN16783257 |
| 364 | VP362 | O3:KUT | YES | 37905 | 35 | 2009 | MT898061 | SAMN16783258 |
| 365 | VP364 | OUT:KUT | YES | 66945 | 64 | 2009 | MT898162 | SAMN16783260 |
| 366 | VP365 | OUT:KUT | YES | 33084 | 32 | 2015 | MT898399 | SAMN16783261 |
| 367 | VP366 | OUT:KUT | YES | 42433 | 40 | 2009 | MT898320 | SAMN16783262 |
| 368 | VP368 | OUT:KUT | YES | 32860 | 30 | 2009 | MT898245 | SAMN16783264 |
| 369 | VP371 | OUT:KUT | YES | 36196 | 34 | 2010 | MT898242 | SAMN16783267 |
| 370 | VP374 | O1:KUT | YES | 33084 | 32 | 2015 | MT898087 | SAMN16783270 |
| 371 | VP379 | O4:KUT | YES | 39074 | 36 | 2015 | MT898359 | SAMN16783274 |
| 372 | VP382 | O11:KUT | YES | 43586 | 40 | 2008 | MT898259 | SAMN16783277 |
| 373 | VP383 | O3:KUT | YES | 39074 | 36 | 2016 | MT898017 | SAMN16783278 |
| 374 | VP385 | OUT:KUT | YES | 36826 | 34 | 2015 | MT898045 | SAMN16783280 |
| 375 | VP386 | O2:KUT | YES | 33766 | 32 | 2010 | MT898388 | SAMN16783281 |
| 376 | VP387 | OUT:KUT | YES | 33602 | 30 | 2009 | MT898375 | SAMN16783282 |
| 377 | VP388 | OUT:KUT | YES | 38891 | 38 | 2007 | MT898280 | SAMN16783283 |
| 378 | VP389 | O3:KUT | YES | 34254 | 35 | 2011 | MT898200 | SAMN16783284 |
| 379 | VP39 | O5:KUT | YES | 33524 | 32 | 2008 | MT898125 | SAMN16782972 |
| 380 | VP393 | O1:KUT | YES | 49023 | 44 | 2008 | MT898338 | SAMN16783288 |
| 381 | VP395 | O3:KUT | YES | 33084 | 32 | 2009 | MT898353 | SAMN16783290 |
| 382 | VP400 | O1:KUT | YES | 37905 | 35 | 2012 | MT898413 | SAMN16783295 |
| 383 | VP403 | OUT:KUT | YES | 33084 | 32 | 2012 | MT898033 | SAMN16783298 |
| 384 | VP408 | O4:KUT | YES | 36826 | 34 | 2015 | MT898334 | SAMN16783302 |
| 385 | VP409 | O4:KUT | YES | 36826 | 34 | 2015 | MT898034 | SAMN16783303 |
| 386 | VP410 | O4:KUT | YES | 39074 | 36 | 2015 | MT898055 | SAMN16783304 |
| 387 | VP411 | O4:KUT | YES | 36826 | 34 | 2015 | MT898089 | SAMN16783305 |
| 388 | VP412 | O4:KUT | YES | 36826 | 34 | 2015 | MT898406 | SAMN16783306 |
| 389 | VP413 | O4:KUT | YES | 36826 | 34 | 2015 | MT898340 | SAMN16783307 |
| 390 | VP414 | O4:KUT | YES | 36826 | 34 | 2015 | MT898071 | SAMN16783308 |
| 391 | VP415 | O4:KUT | YES | 36826 | 34 | 2015 | MT898333 | SAMN16783309 |
| 392 | VP417 | O4:KUT | YES | 39074 | 36 | 2016 | MT898106 | SAMN16783311 |
| 393 | VP418 | O4:KUT | YES | 39074 | 36 | 2016 | MT898171 | SAMN16783312 |
| 394 | VP419 | O4:KUT | YES | 36826 | 34 | 2016 | MT898249 | SAMN16783313 |
| 395 | VP420 | O4:KUT | YES | 39074 | 36 | 2016 | MT898335 | SAMN16783314 |
| 396 | VP422 | O4:KUT | YES | 39074 | 36 | 2016 | MT898416 | SAMN16783316 |
| 397 | VP423 | OUT:KUT | YES | 39074 | 36 | 2016 | MT898374 | SAMN16783317 |
| 398 | VP424 | O3:KUT | YES | 39074 | 36 | 2016 | MT898131 | SAMN16783318 |
| 399 | VP425 | O4:KUT | YES | 36826 | 34 | 2016 | MT898247 | SAMN16783319 |
| 400 | VP426 | O4:KUT | YES | 36826 | 34 | 2016 | MT898287 | SAMN16783320 |
| 401 | VP427 | O4:KUT | YES | 36826 | 34 | 2016 | MT898256 | SAMN16783321 |
| 402 | VP429 | O4:KUT | YES | 30795 | 29 | 2017 | MT898050 | SAMN16783323 |
| 403 | VP430 | O4:KUT | YES | 39074 | 36 | 2017 | MT898349 | SAMN16783324 |
| 404 | VP431 | O4:KUT | YES | 39074 | 36 | 2017 | MT898318 | SAMN16783325 |
| 405 | VP432 | O4:KUT | YES | 39074 | 36 | 2017 | MT898315 | SAMN16783326 |
| 406 | VP433 | O4:KUT | YES | 35555 | 34 | 2008 | MT898220 | SAMN16783327 |
| 407 | VP435 | O4:KUT | YES | 39074 | 36 | 2017 | MT898111 | SAMN16783329 |
| 408 | VP436 | O4:KUT | YES | 36826 | 34 | 2017 | MT898230 | SAMN16783330 |
| 409 | VP437 | O4:KUT | YES | 39074 | 36 | 2017 | MT898098 | SAMN16783331 |
| 410 | VP438 | O4:KUT | YES | 39074 | 36 | 2017 | MT898248 | SAMN16783332 |
| 411 | VP445 | O4:KUT | YES | 36826 | 34 | 2017 | MT898381 | SAMN16783339 |
| 412 | VP446 | O1:KUT | YES | 28239 | 26 | 2017 | MT898209 | SAMN16783340 |
| 413 | VP46 | O2:KUT | YES | 79854 | 72 | 2010 | MT898088 | SAMN16782978 |
| 414 | VP47 | O2:KUT | YES | 79855 | 72 | 2010 | MT898268 | SAMN16782979 |
| 415 | VP5 | O3:KUT | YES | 38016 | 37 | 2010 | MT898276 | SAMN16782948 |
| 416 | VP52 | OUT:KUT | YES | 36288 | 36 | 2010 | MT898049 | SAMN16782984 |
| 417 | VP62 | O1:KUT | YES | 31301 | 30 | 2006 | MT898239 | SAMN16782993 |
| 418 | VP68 | O4:KUT | YES | 31035 | 32 | 2014 | MT898137 | SAMN16782997 |

The data about 18 rare *V.parahaemolyticus* K-serogroups isolates that support the findings of this study can be found in the GenBank database (https://www.ncbi.nlm.nih.gov/). The accession numbers are listed in the Table S9.

| **TABLE S9** \| The accession numbers of 18 rare *V.parahaemolyticus* strains. | |
| --- | --- |
| **Serotype** | **Accession number** |
| K7 | MK455080 |
| K15 | MK455086 |
| K22 | MK463648 |
| K24 | MK482098 |
| K39 | MK473651 |
| K40 | MK473650 |
| K43 | MK473647 |
| K45 | MK473645 |
| K46 | MK473644 |
| K51 | MK473658 |
| K52 | MK473659 |
| K53 | MK473660 |
| K54 | MK482084 |
| K59 | MK482088 |
| K64 | MK482091 |
| K65 | MK482092 |
| K67 | MK482094 |
| K70 | MK482097 |
